# ^19^F MRI/CEUS Dual Imaging‐Guided Sonodynamic Therapy Enhances Immune Checkpoint Blockade in Triple‐Negative Breast Cancer

**DOI:** 10.1002/advs.202401182

**Published:** 2024-07-25

**Authors:** Qiu Chen, Hong Xiao, Lijun Hu, Yongquan Huang, Zhong Cao, Xintao Shuai, Zhongzhen Su

**Affiliations:** ^1^ Department of Ultrasound The Fifth Affiliated Hospital Sun Yat‐Sen University Zhuhai 519000 P. R. China; ^2^ Department of Medical Ultrasonic The Third Affiliated Hospital of Sun Yat‐sen University Guangzhou 510630 P. R. China; ^3^ School of Biomedical Engineering Shenzhen Campus of Sun Yat‐sen University Shenzhen Guangdong 518107 P. R. China; ^4^ Shenzhen International Institute for Biomedical Research Longhua District Shenzhen Guangdong 518116 P. R. China; ^5^ Nanomedicine Research Center The Third Affiliated Hospital of Sun Yat‐sen University Guangzhou 510630 P. R. China

**Keywords:** ^19^F magnetic resonance imaging, bimodal imaging, contrast‐enhanced ultrasound, sonodynamic therapy, triple‐negative breast cancer

## Abstract

Treatment of highly aggressive triple‐negative breast cancer (TNBC) in the clinic is challenging. Here, a liposome nanodrug (LP@PFH@HMME) integrating imaging agents and therapeutic agents for bimodal imaging‐guided sonodynamic therapy (SDT) is developed, which boosted immunogenicity to enable potent immunotherapy via immune checkpoint blockade (ICB) in TNBC. In the acidic tumor microenvironment (TME), LP@PFH@HMME undergoes “nano‐to‐micro” transformation due to a pH‐responsive lipid fusion, which makes droplets much more sensitive to ultrasound (US) in contrast‐enhanced ultrasound (CEUS) and SDT studies. The nanodrug demonstrates robust bimodal imaging ability through fluorine‐19 magnetic resonance imaging (^19^F MRI) and CEUS bimodal imaging, and it exhibits excellent solubility in aqueous solution with relatively high ^19^F content and desirable long transverse relaxation time (T_2_ = 1.072 s), making it suitable for high‐performance ^19^F MRI, in addition to effective accumulation of nanodrugs after tail vein injection. Thus, ^19^F MRI/CEUS dual imaging is achievable to show adequate time points for US irradiation of tumor sites to induce highly effective SDT, which produces abundant reactive oxygen species (ROS) triggering immunogenic cell death (ICD) to assist ICB‐based immunotherapy. The combination treatment design of sonodynamic therapy with immunotherapy effectively inhibited TNBC growth and recurrence, highlighting the promise of multifunctional nanodrugs in treating TNBC.

## Graphical abstract

A tumor microenvironment‐sensitive liposome nanomedicine that integrates imaging agents and therapeutic agents for ^19^F MR/CEUS bimodal imaging‐guided sonodynamic therapy aimed at enhancing immune checkpoint blockade and effectively inhibiting triple‐negative breast cancer growth and recurrence.

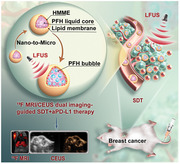

## 1 Introduction

Triple‐negative breast cancer (TNBC) is a worldwide prevalent malignancy characterized by a poor prognosis.^[^ ^1^ ^]^ The highly invasive nature of TNBC is underscored by the fact that ≈46% of patients have distant metastasis. Tragically, the median survival time postmetastasis is a mere 13.3 months, and the unsettling recurrence rate postsurgery soars to a staggering 25%.^[^ ^2^ ^]^ Moreover, highly efficient and standardized TNBC treatment regimens are still lacking.^[^ ^3^ ^]^ In recent years, immunotherapy based on immune checkpoint blockade (ICB),^[^ ^4^, ^5^, ^6^ ^]^ typical including anti‐CTLA4 therapy^[^ ^7^, ^8^ ^]^ and PD‐L1/PD‐1 therapy,^[^ ^9^, ^10^, ^11^, ^12^ ^]^ has shown promising outcomes as an innovative therapeutic approach across various cancers. Unfortunately, only a subset of TNBC patients exhibit efficient antitumor responses to ICB therapy,^[^ ^13^ ^]^ with response rates of ≈5% for unselected patients and ≈23% for treatment‐naive PD‐L1‐positive patients treated with pembrolizumab.^[^ ^14^ ^]^ Therefore, there exists an urgent need to develop new strategies to achieve substantial therapeutic benefits via effectively enhancing immune responses in TNBC patients.^[^ ^15^ ^]^ Currently, promising therapeutic approaches, such as chemotherapy,^[^ ^15^ ^]^ radiotherapy^[^ ^16^, ^17^, ^18^ ^],^ photodynamic therapy (PDT),^[^ ^19^ ^]^ and sonodynamic therapy (SDT) in combination with ICB, have demonstrated synergistic antitumor effects.^[^ ^20^ ^]^ Among these approaches, SDT, a form of ultrasound‐mediated therapeutic technology, has drawn considerable attention as a potential approach for cancer therapy.^[^ ^21^, ^22^ ^]^ SDT can induce immunogenic cell death (ICD) in cancer cells by generating reactive oxygen species (ROS),^[^ ^23^, ^24^ ^]^ leading to increased tumor antigen exposure and promoting dendritic cells (DCs) antigen presentation. This, in turn, initiates an antitumor immune cascade response to enhance the efficacy of ICB therapy.^[^ ^23^, ^24^ ^]^

Recently, imaging‐guided therapy has emerged as a promising approach for enhancing therapeutic efficacy.^[^ ^25^, ^26^, ^27^, ^28^ ^]^ First, imaging facilitates the assessment of tumor characteristics such as size, shape, location, and their relation to surrounding tissues, aiding in determining treatment position and scope. Second, imaging enables the selection of an optimal treatment time by monitoring the arrival of contrast agents at the targeted lesion. Third, posttherapy disease progression can be promptly monitored through imaging. Among the various imaging techniques for breast cancer, proton magnetic resonance imaging (^1^H MRI) is widely utilized due to its ability to penetrate deep tissue, high spatial resolution, and non‐invasiveness.^[^ ^29^ ^]^ However, ^1^H MRI faces challenges in detecting subtle differences due to high biological background noise.^[^ ^30^ ^]^ A promising alternative is fluorine‐19 magnetic resonance imaging (^19^F MRI), which leverages 100% of the natural abundance and relatively high sensitivity (83% of protons) of the ^19^F nucleus.^[^ ^31^, ^32^ ^] 19^F MRI is a potent technique for in vivo molecular imaging and clinical diagnosis because of its negligible background noise due to the short spin–spin relaxation time of fluorine atoms.^[^ ^33^ ^]^ However, ^19^F MRI lacks real‐time information. Consequently, the rapid development of multimodal imaging techniques, which integrate the advantages of each imaging modality, has gained prominence. These techniques offer complementary information about cancerous conditions, enabling precise and efficient personalized cancer treatment.^[^ ^34^, ^35^ ^]^ Several multimodal imaging techniques, including ^19^F MR/fluorescence imaging (FLI),^[^ ^25^ ^] 19^F MR/photoacoustic imaging (PAI)^[^ ^26^ ^]^ and ^19^F MR/near‐infrared fluorescence imaging (NIR FLI),^[^ ^27^ ^]^ have been reported in recent years to extend the application scope of ^19^F MRI. Among the various imaging modalities available, the integration of ^19^F MRI with contrast‐enhanced ultrasound (CEUS) has significant value and utility. CEUS, a widely used and safe technique for diagnosing breast cancer in clinical practice, has been extensively explored for tumor imaging due to its non‐invasiveness, favorable tissue penetration, cost‐effectiveness, and nonionizing properties.^[^ ^36^, ^37^, ^38^ ^]^ CEUS is a convenient imaging technique that provides real‐time images with high sensitivity. However, effective monitoring of deep organs is challenging. Therefore, the combination of ^19^F MRI with CEUS can yield quantitative high‐contrast “hot spot” images with unlimited tissue penetration depth and minimal background signals. Importantly, integrating ^19^F MRI/CEUS dual imaging‐guided synergistic therapy may show significant potential in enhancing the precision and efficacy of TNBC treatment.

To optimize the effectiveness of ultrasonography, traditional ultrasonic contrast agents, such as SonoVue, are typically microsized.^[^ ^39^ ^]^ However, these microbubbles, which exhibit enhanced echo contrast on ultrasound, often encounter challenges in penetrating the leaky vasculature of tumors.^[^ ^40^, ^41^ ^]^ Regrettably, for effective cancer therapy, nanodrugs ideally require a relatively small size (100–400 nm) to facilitate prolonged blood circulation and accumulation in tumors through the enhanced permeability and retention (EPR) effect.^[^ ^42^ ^]^ Therefore, developing a smart nano platform that undergoes spontaneous “nano micro” growth in response to the tumor microenvironment (TME) would be a highly beneficial strategy for solving the dilemma of imaging and therapy in one nano drug delivery system.^[^ ^38^ ^]^ For instance, the nanodrug could be designed to undergo a “ nano‐to‐micro” transformation within the acidic TME due to the pH‐responsive lipid fusion of cholesterol (Chol).^[^ ^38^, ^43^ ^]^ For dual imaging, perfluorocarbons have recently emerged as valuable agents for both ^19^F MRI and CEUS.^[^ ^38^, ^44^ ^]^ Among these, perfluorohexane (PFH) lipids offer relatively high stability in vivo due to their elevated boiling point (≈56 °C).^[^ ^45^ ^]^ Once under US irradiation, the PFH lipid rapidly gasifies, producing a cavitation force for CEUS imaging.

**Figure 1:**
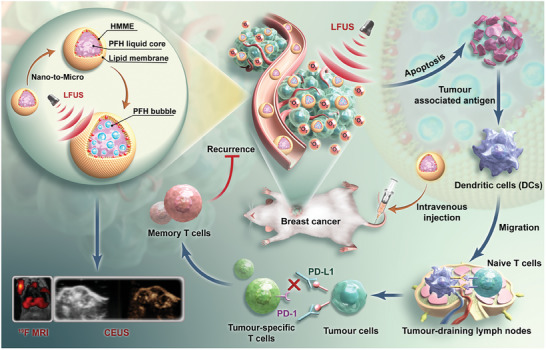
Schematic illustration of ^19^F MR/CEUS dual imaging‐guided sonodynamic therapy enhancing immune checkpoint blockade of triple‐negative breast cancer.

Herein, we developed a liposome nano‐drug that integrates both an imaging agent and a therapeutic agent, offering a dual‐modality for imaging‐guided SDT and enhancing ICB for TNBC (**Figure** **1**). Our approach involved encapsulating the sonosensitizer haematoporphyrin monomethyl ether (HMME) and the ^19^F MRI/CEUS probe perfluorohexane (PFH, boiling point ≈56 °C) within the nanodrug, denoted as LP@PFH@HMME. The incorporation of PFH within LP@PFH@HMME was expected to endow the system with robust bimodal imaging capacity, particularly in ^19^F MRI and CEUS. Subsequent SDT was initiated, where the sonosensitizer HMME generated ROS under US irradiation, targeting TNBC cells. This SDT‐induced tumor cell ICD may further trigger antitumor immune activation by facilitating DCs maturation. Under US irradiation, LP@PFH@HMME may exhibit a significant antitumor effect, and its impact was further potentiated when combined with PD‐L1 checkpoint blockade using anti‐PD‐L1 antibody (aPD‐L1), resulting in long‐term immune memory dysfunction. In vitro and in vivo experiments were conducted to explore the potential of LP@PFH@HMME in inducing SDT and ICB‐based immunotherapy.

## 2 Results and Discussion

### 2.1 Characterization of LP@PFH@HMME

**Figure 2:**
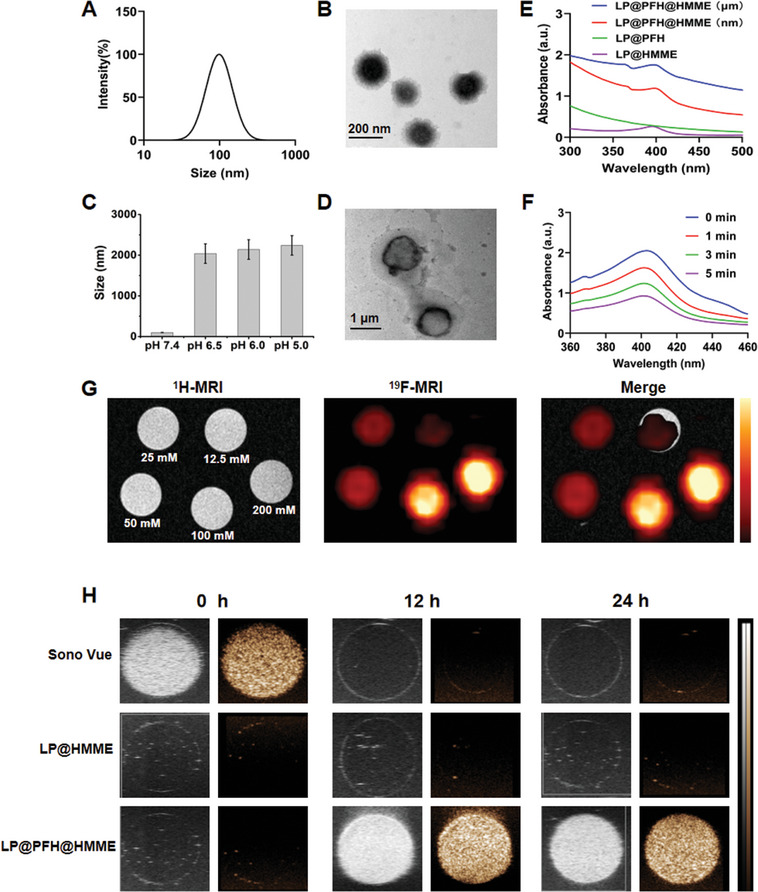
Characterization of the nanodrug. A) Particle sizes and B) TEM image of LP@PFH@HMME at pH 7.4. (*n* = 3). C) “Nano‐to‐micro” transformation at 37 °C and different pH values (*n* = 3). D) TEM image of LP@PFH@HMME after incubation at pH 6.5 for 24 h. E) UV–vis absorption spectra of different nanodrug formulations. F) Time‐dependent DPBF absorption spectra in the presence of LP@PFH@HMME under US irradiation for different durations. G) ^1^H/^19^F MR images of LP@PFH@HMME at various concentrations. H) US imaging capacity of LP@PFH@HMME after growing to microbubbles under US irradiation (1.0 MHz, 1.6 W cm^−2^, 50% duty cycle, 5 min). SonoVue and LP@HMME were used as controls. (Left, grayscale; right, harmonic in each pair of images).

Liposomes were employed as the nano platform for encapsulating the hydrophobic sonosensitizer HMME and PFH liquid. The resulting PFH‐encapsulated/HMME‐loaded nanodrug (LP@PFH@HMME) exhibited excellent dispersion in a pH 7.4 PBS solution and demonstrated favorable stability over a span of one week at 4 °C, as illustrated in Figures S1 and S2A (Supporting Information). Characterized by dynamic light scattering (DLS), LP@PFH@HMME displayed an average diameter of 105 ± 1.64 nm (PDI = 0.198 ± 0.022) (**Figure** **2**A). Transmission electron microscopy (TEM) images showed that the LP@PFH@HMME particles appeared as quasispheres (Figure 2B). The “nano‐to‐micro” size expansion of the nanodrug has been shown to enhance its US imaging capacity. It has been reported that cholesterol (Chol) may disrupt the stability of phosphatidylethanolamine (PE) and phosphocholine (PC)‐PE bilayers, leading to lipid fusion. Moreover, acidic conditions can facilitate this lipid fusion process.^[^ ^45^ ^]^ As illustrated in Figures 2C and S2B (Supporting Information), the size of the LP@PFH@HMME particles increased from ≈100 nm to more than 2 µm after a 24‐h incubation in acidic conditions (i.e., pH 6.5, pH 6.0, and pH 5.0). The TEM image in Figure 2D further demonstrates the transformation of the initially nanosized LP@PFH@HMME nanoparticles into microsized particles after incubation at pH 6.5 for 24 h. These findings confirm the ability of the nanodrug to promote “nanophotochemical” transformation in the acidic tumor microenvironment. UV–vis analysis revealed a distinctive absorption peak at 398 nm, attributed to HMME, confirming the successful encapsulation of HMME in LP@PFH@HMME (Figure 2E). As verified in Figure S3 (Supporting Information), 44.97% ± 2.56% of the HMME was released after 24 h at pH 6.4, indicating that HMME would leak from liposomes during the fusion process in a slightly acidic environment. The encapsulation efficiency of HMME in LP@PFH@HMME was 83.79% based on the standard absorption curve of HMME (Figure S4, Supporting Information). Additionally, under US irradiation, 1,3‐diphenylisobenzofuran (DPBF) quenching induced by singlet oxygen (^1^O_2_) from LP@PFH@HMME suggested a potential SDT effect against tumors (Figure 2F).

Furthermore, LP@PFH@HMME exhibited a robust signal at −83.97 ppm, corresponding to the highest signal‐to‐noise ratio (SNR) in ^19^F nuclear magnetic resonance (NMR) (Figure S5, Supporting Information). The ^19^F NMR SNR of the nanodrug demonstrated a linear correlation with the PFH concentration (25–400 mm, R^2^ = 0.9996) (Figure S6, Supporting Information). This broad fitting range, even at high concentrations of LP@PFH@HMME, holds promising potential for ^19^F MR imaging. The relaxation times (T_1_ and T_2_) of ^19^F nuclei are critical factors influencing the sensitivity of ^19^F MRI probes.^[^ ^45^ ^]^ Generally, short T_1_ and long T_2_ are advantageous for achieving high ^19^F MRI signal intensity. Notably, LP@PFH@HMME exhibited a favorable longitudinal relaxation time (T_1_ = 1.756 s) and transverse relaxation time (T_2_ = 1.072 s), indicating a robust ^19^F MRI signal. To accurately assess the PFH encapsulation efficiency, we employed quantitative Nuclear Magnetic Resonance (qNMR) to measure the number of loaded fluorine nuclei. Utilizing CF_3_COOH as an internal reference, we adopted the AQARI method for precise quantitation. The results revealed an encapsulation efficiency of a remarkable 58.57%, demonstrating the high performance of our encapsulation technique. Figure 2G,H illustrates the effective bimodal imaging capacity of LP@PFH@HMME on ^19^F MRI and contrast‐enhanced ultrasound (CEUS). The ^19^F MRI (Figure 2G) and CEUS (Figure S7, Supporting Information) signals exhibited a progressive increase with increasing PFH concentration. To assess the ability of LP@PFH@HMME to perform sustained CEUS imaging and power Doppler (PD) imaging after microbubble formation under US irradiation (1.0 MHz, 1.6 W cm^−2^, 50% duty cycle, 5 min), SonoVue and LP@HMME were utilized as controls. Notably, LP@PFH@HMME demonstrated an extended imaging window. There was no significant difference in ultrasound signal intensity at 12 h or 24 h post‐US exposure, in contrast to the much narrower time window observed for SonoVue (Figure 2H; Figure S8, Supporting Information).

### 2.2 In Vitro Cellular Internalization

**Figure 3:**
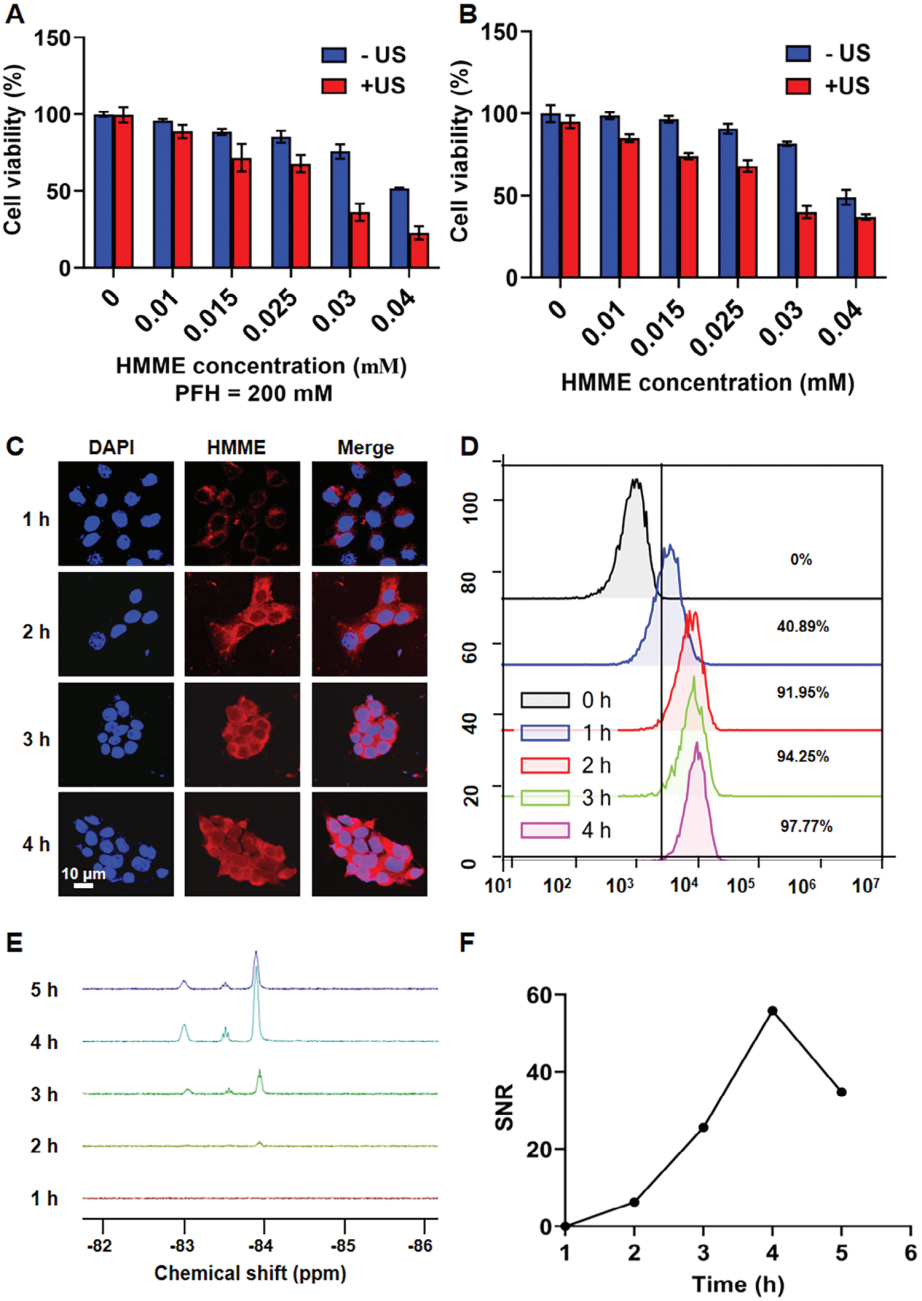
Cellular internalization of the nanodrug. Cell viability of 4T1 cells incubated with A) LP@PFH@HMME or B) LP@HMME for 12 h at various concentrations with/without US irradiation (US, 1.0 MHz, 1.6 W cm^−2^, 50% duty cycle, 5 min) (*n* = 3). Cellular uptake of LP@PFH@HMME in 4T1 cells observed by CLSM C) and analyzed by flow cytometry D). E) ^19^F nuclear magnetic resonance (NMR) spectra signal intensity and F) signal‐to‐noise ratio (SNR) of 4T1 cells after incubation with LP@PFH@HMME for different durations.

The efficacy of SDT‐induced cancer cell killing was evaluated using a Cell Counting Kit‐8 (CCK‐8) assay. After US irradiation, 4T1 cells incubated with LP@PFH@HMME (**Figure** **3**A) or LP@HMME (Figure 3B) exhibited a viability ˂40% at the HMME concentration of 0.03 mm, indicating a highly efficient SDT effect. Notably, cell viability remained above 90% irrespective of US irradiation, even at LP@PFH concentrations up to 400 mm, suggesting negligible cytotoxicity of PFH against 4T1 cells (Figure S9, Supporting Information). The cellular uptake of LP@PFH@HMME by 4T1 cells was assessed using confocal laser scanning microscopy (CLSM) and flow cytometry. CLSM images revealed the efficient internalization of LP@PFH@HMME by 4T1 cells, with a noticeable increase in cellular uptake within the initial 2 h (Figure 3C). Flow cytometry analysis demonstrated a high intracellular uptake efficiency, reaching 97.77% after 4 h of incubation (Figure 3D), consistent with the CLSM observations. Further confirmation of cellular internalization was conducted using ^19^F NMR. The results indicated a gradual increase in the ^19^F NMR signal intensity of the cell lysates, which peaked at 4 h (Figure 3E,F). The efficient intracellular uptake of LP@PFH@HMME in 4T1 cells facilitated subsequent ^19^F magnetic resonance (MR)/CEUS dual imaging and intracellular SDT for further in vivo studies.

### 2.3 In Vitro ROS Generation and DCs Maturation

**Figure 4:**
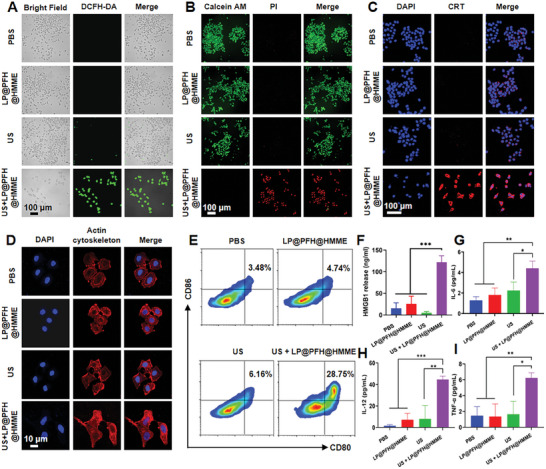
In vitro ROS generation by LP@PFH@HMME in 4T1 cells upon US irradiation and DCs maturation. A) CLSM images of 4T1 cells showing ROS generation detected by DCFH‐DA and B) CLSM images of 4T1 cells receiving different treatments stained with calcein‐AM and PI (scale bar = 100 µm). C) CRT exposure after different treatments (scale bar = 100 µm). D) CLSM images of DC2.4 cells after different treatments. The cytoskeleton was stained with phalloidin (red), and the nucleus was stained with DAPI (blue) (scale bar = 10 µm). E) A typical flow cytometry assay of mature BMDCs (CD80^+^CD86^+^). F) HMGB1 release in 4T1 cells after different treatments was measured via ELISA (*n* = 3). G–I) Quantification of the secretion of IL‐6 (G), IL‐12 (H), and TNF‐α (I) in DCs suspensions (*n* = 3). ^*^ *p* < 0.05, ^**^ *p* < 0.01, ^***^ *p* < 0.001.

To elucidate the SDT mechanism of LP@PFH@HMME under US irradiation in 4T1 cells, the intracellular ROS levels were assessed using 2′‐7′‐dichlorofluorescein diacetate (DCFH‐DA). **Figure** **4**A shows that cells treated with US + LP@PFH@HMME exhibited a substantial increase in intracellular ROS and the flow cytometry data showed that >95% of the cells were ROS positive (Figure S10, Supporting Information). In contrast, neither LP@PFH@HMME (HMME, 0.03 mm) without US irradiation nor US irradiation alone had a discernible impact on ROS production. Furthermore, the therapeutic efficacy of the nanodrug was visualized through calcein‐AM/PI staining via CLSM. Figure 4B shows that 4T1 tumor cells treated with US + LP@PFH@HMME exhibited intense red fluorescence, indicating that LP@PFH@HMME effectively induced SDT under US irradiation, leading to cancer cell death. SDT‐induced ICD leads to the exposure of 4T1 cells to calreticulin (CRT), which acts as an “eat me” signal to trigger the maturation of DCs.^[^ ^49^ ^]^ Treatment with the SDT augmented by LP@PFH@HMME substantially induced CRT exposure, as indicated by red fluorescence, indicating an efficient ICD signal under the SDT conditions (Figure 4C). Subsequently, the maturation of DCs induced by ICD was evaluated. As depicted in Figure 4D, DC2.4 cells treated with US + LP@PFH@HMME exhibited a noticeable dendritic structure, enhancing the presentation of cancer antigens. No discernible morphological differences were observed in DC2.4 cells between the US and LP@PFH@HMME treatment groups and the PBS group. Furthermore, the maturation of DCs (CD80^+^CD86^+^) was quantified using flow cytometry. The augmented SDT treatment with LP@PFH@HMME significantly promoted DCs maturation, with the proportion of mature DCs in the US + LP@PFH@HMME treatment group being 4.63 times higher than that in the PBS group (Figure 4E; Figure S11, Supporting Information). Furthermore, the key biomarker of ICD, high mobility group box 1 (HMGB‐1), was assessed using an enzyme‐linked immunosorbent assay (ELISA). Figure 4F shows that the HMGB‐1 concentrations in the groups treated with US and LP@PFH@HMME were comparable to the levels observed in the PBS group. In contrast, the concentration of HMGB‐1 increased significantly after exposure to US + LP@PFH@HMME, reaching a level 8 times greater than that in the PBS group. Cytokine secretion is a well‐documented phenomenon in the immune response. Consistent with the observed maturation of DCs, remnants of 4T1 tumor cells following SDT augmented by LP@PFH@HMME could trigger heightened immune cytokine secretion from DCs. These changes included notable increases in IL‐6, as depicted in Figure 4G; IL‐12, as illustrated in Figure 4H; and TNF‐α, as shown in Figure 4I. The abovementioned results confirmed that the augmentation of SDT by LP@PFH@HMME induces robust ICD. This is evident through amplified CRT exposure and heightened HMGB‐1 levels. These molecular events culminate in a substantial enhancement of DCs) maturation.

### 2.4 In Vivo ^19^F MRI and US imaging

BALB/c mice (female, 4–6 weeks old) were provided by Guangdong Gempharmatech Co., Ltd. The animal use protocol used in this study was reviewed and approved by the Institutional Animal Care and Use Committee (IACUC) of Sun Yat‐Sen University (SYSU‐IACUC‐2023‐000641). To establish the breast tumor model, 1 × 10^6^ of 4T1 cells suspended in 100 µL of PBS were injected subcutaneously into the right breast of each mouse. After 7 days, the breast tumors were measured; the tumors were allowed to reach ≈100 mm^3^ in size.

**Figure 5:**
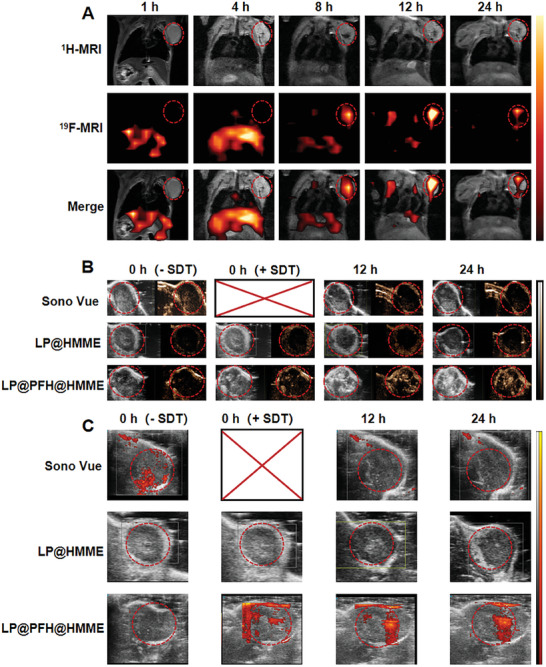
In vivo ^1^H/^19^F MRI and US imaging. In vivo, ^1^H/^19^F MRI A), CEUS B), and PD C) images of the tumor‐bearing mice at different times after intravenous injection of LP@PFH@HMME (200 mm, 100 µL).

The presence of perfluorohexane (PFH) within LP@PFH@HMME enables ^19^F MR/CEUS dual imaging capabilities. **Figure** **5**A and ^19^F MRI images reveal the effective accumulation of LP@PFH@HMME in the tumor region, where it reached its peak concentration at 12 h after intravenous injection. By integrating anatomical data obtained from ^1^H MRI, ^19^F MRI generated “hot‐spot” images, providing visual insights into the in vivo biodistribution of LP@PFH@HMME. Concurrently, DiR fluorescence imaging corroborated these findings, demonstrating that LP@PFH@HMME exhibited maximum accumulation at the tumor site around the 12‐h mark (Figure S12, Supporting Information). Consequently, the optimal time for SDT was 12 h after the intravenous injection of the nanodrugs, which was consistent with the time‐dependent biodistribution pattern of LP@PFH@HMME.

To assess the US imaging capability of LP@PFH@HMME, CEUS and power Doppler (PD) images were recorded following intravenous injection of SonoVue, LP@HMME, or LP@PFH@HMME into tumor‐bearing mice. As depicted in Figure 5B,C, LP@PFH@HMME exhibited a broad time window for US imaging. Robust US signal intensities were observed at both 12 and 24 h after intravenous injection through the tail vein under US exposure conditions (1.0 MHz, 1.6 W cm^−2^, 50% duty cycle, 5 min). These findings underscore the favorable retention properties of LP@PFH@HMME for ultrasonographic detection, which holds significance for guiding subsequent SDT.

### 2.5 In Vivo Antitumor Therapeutic Efficacy

**Figure 6:**
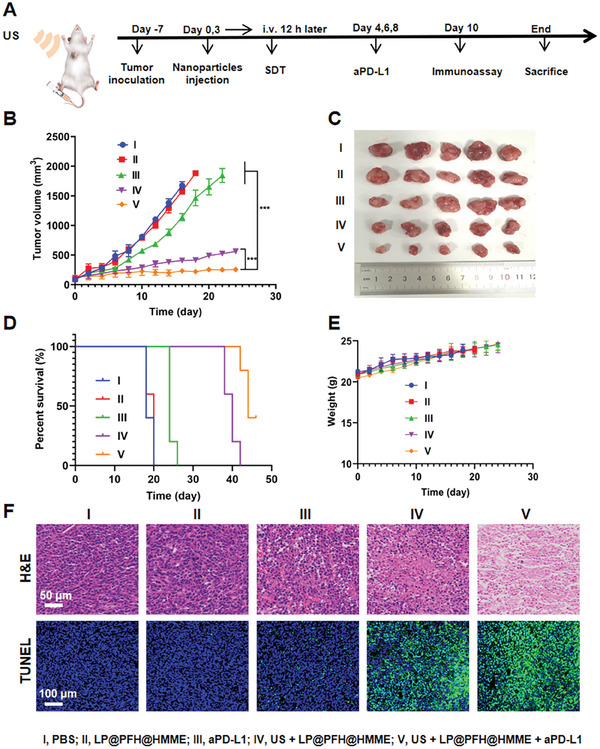
In vivo antitumor therapeutic efficacy of LP@PFH@HMME‐based sonodynamic immunotherapy. A) Schematic illustration of the animal experimental design. B) tumor growth curves against time for 4T1 tumor‐bearing mice receiving different treatments (*n* = 5, ^***^ *p* < 0.001). C) Photograph of tumors excised from 4T1 tumor‐bearing mice receiving different treatments. D) The cumulative survival of mice receiving different treatments (*n* = 5). E) Changes in the body weights of 4T1 tumor‐bearing mice receiving different treatments showing negligible side effects (*n* = 5). F) Histological analysis of 4T1 tumor tissues via H&E staining (scale bar: 50 µm) and TUNEL staining (scale bar: 100 µm).

Encouraged by promising in vitro results demonstrating antitumor efficacy and immune activation through ICD, we expanded our investigation to evaluate the in vivo antitumor effects of LP@PFH@HMME‐based SDT and its combination with ICB in 4T1 tumor‐bearing mice. The mice were randomly assigned to five groups: PBS, LP@PFH@HMME, aPD‐L1, US + LP@PFH@HMME, and US + LP@PFH@HMME + aPD‐L1. Seven days after tumor inoculation, when the tumor volume reached ≈100 mm^3^, the first dose of each formulation was administered via intravenous tail vein injection, followed by subsequent doses every 3 days. US irradiation was applied 12 h after each intravenous injection, and in the aPD‐L1 and US + LP@PFH@HMME + aPD‐L1 groups, the aPD‐L1 antibody was intraperitoneally administered on days 4, 6, and 8 (**Figure** **6**A). As depicted in Figure 6B,C, tumor growth was significantly suppressed in the aPD‐L1 monotherapy treatment group, in contrast with the rapid growth observed in the PBS and LP@PFH@HMME groups. Notably, US + LP@PFH@HMME treatment markedly inhibited tumor growth, emphasizing the specificity of the therapeutic effect of LP@PFH@HMME under US irradiation. Interestingly, the combination therapy of US + LP@PFH@HMME + aPD‐L1 exhibited the most potent tumor growth inhibition effect. The results of the survival analysis were consistent with the findings on tumor growth inhibition. Despite both the US + LP@PFH@HMME and US + LP@PFH@HMME + aPD‐L1 groups demonstrating favorable effects on mouse survival, the US + LP@PFH@HMME + aPD‐L1 treatment appeared to be the most effective at prolonging animal survival (Figure 6D). Furthermore, there were no significant changes in the body weights of the mice across all treatment groups (Figure 6E).

Haematoxylin‐eosin (H&E) images of major organs revealed no apparent pathological signs of systemic toxicity (Figure S13, Supporting Information). The serum biochemistry indices did not significantly differ between the treatment groups and the PBS group (Figure S14, Supporting Information). These results underscore the high therapeutic biosafety of combination therapy involving nanodrug‐based SDT and immunotherapy. The synergistic therapeutic efficacy of nanodrug‐based SDT and immunotherapy was further substantiated through histopathological assessments. Examination of tumor tissues via H&E staining and TUNEL staining revealed the highest proportion of apoptotic and necrotic cells in the US + LP@PFH@HMME + aPD‐L1 treatment group, indicative of the remarkable therapeutic impact achieved through the combined treatment approach of SDT and ICB (Figure 6F).

### 2.6 In Vivo Antitumor Immune Activation

**Figure 7:**
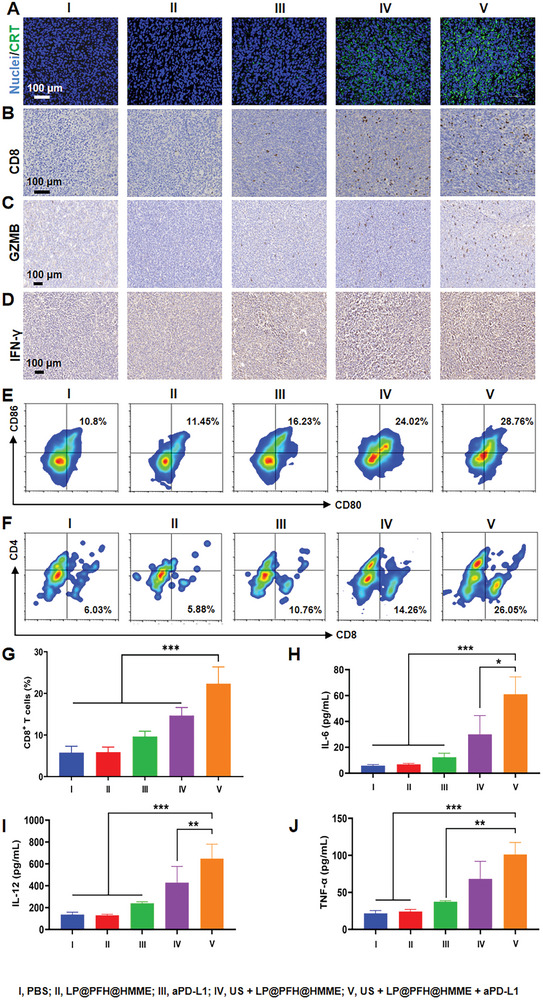
In vivo, LP@PFH@HMME‐augmented SDT enhanced aPD‐L1 therapy‐induced systemic antitumor immunity. A) CRT immunofluorescence staining and immunohistochemical (IHC) staining of B) CD8^+^ T cells, C) GZMB^+^ cells, and D) IFN‐γ^+^ cells in 4T1 tumor tissues (scale bar: 100 µm). Flow cytometry analysis of E) mature DCs (gated on CD11c^+^ cells) within lymph nodes and F) CD8^+^ T cells (gated on CD3^+^) within tumor tissues. G) Quantitative statistics of CD8^+^ T cells in different treatment groups (*n* = 5). H–J) Intratumoral levels of IL‐6, IL‐12, and TNF‐α after different treatments (*n* = 5). ^*^ *p* < 0.05, ^**^ *p* < 0.01, ^***^ *p* < 0.001.

To explore the underlying immune activation of the antitumor effects triggered by LP@PFH@HMME‐based SDT combined with PD‐L1 blockade, immune cells in tumors and lymph nodes (LNs) were collected, and their immunophenotypes were analyzed. As shown in **Figure** **7**A, 4T1 tumor tissues from mice treated with PBS, LP@PFH@HMME, or aPD‐L1 alone exhibited minimal calreticulin (CRT) exposure (green fluorescence), whereas US + LP@PFH@HMME and US + LP@PFH@HMME + aPD‐L1 treatments induced evident CRT exposure, signifying an efficient ICD signal under SDT. Subsequently, ICD‐induced DCs maturation in 4T1 tumor‐bearing mice was assessed via flow cytometry.

To further assess the antitumor immune effect, T‐cell activation within tumor tissues was explored. Immunohistochemical staining, flow cytometry assays, and immunofluorescence staining collectively demonstrated that CD8^+^ T cells were scarcely present in the tumor tissues of the PBS and LP@PFH@HMME treatment groups. In contrast, the groups treated with US + LP@PFH@HMME or US + LP@PFH@HMME + aPD‐L1 exhibited significant increases in the percentage of tumor‐infiltrating CD8^+^ T cells, reaching proportions of 14.67% and 22.34%, respectively (Figure 7B,F,G). These findings highlight the substantial enhancement in T‐cell infiltration within the tumor microenvironment in response to combined treatment involving sonodynamic therapy and PD‐L1 blockade.

It is widely recognized that the anti‐PD‐L1 antibody enhances antitumor immunity against CD8^+^ T cells by stimulating the production of tumor cell‐killing factors, such as GZMB (granzyme B) and IFN‐γ (interferon‐gamma).^[^ ^50^, ^51^ ^]^ In comparison to other treatments (i.e., PBS, LP@PFH@HMME, aPD‐L1, and US + LP@PFH@HMME), US + LP@PFH@HMME + aPD‐L1 treatment resulted in the highest levels of GZMB and IFN‐γ within the tumor (Figure 7C,D). In the lymph nodes, the number of mature DCs (CD11c^+^CD80^+^CD86^+^) in the US + LP@PFH@HMME treatment group was 2.15 times greater than that in the PBS group, suggesting that LP@PFH@HMME‐based SDT induces ICD to enhance DCs maturation. Remarkably, the number of mature DCs in the US + LP@PFH@HMME + aPD‐L1 group further increased compared to that in the US + LP@PFH@HMME group, reaching 2.5 times that of the PBS group. These findings emphasized that aPD‐L1 could further promote DCs activation (Figure 7E; Figure S15, Supporting Information). Within the tumor tissue samples, the US + LP@PFH@HMME + aPD‐L1 group displayed a 1.5‐fold increase in the number of tumor‐infiltrating CD8^+^ T cells compared to that in the PBS group (Figure 7G). These findings indicate that combined therapy comprising SDT and anti‐PD‐L1 ICB enhances the activation of T cells. Furthermore, in both the US + LP@PFH@HMME and US + LP@PFH@HMME + aPD‐L1 groups, there was a notable increase in cytokine levels (including IL‐6, IL‐12, and TNF‐α) within the tumor tissue of 4T1 tumor‐bearing mice. However, the US + LP@PFH@HMME + aPD‐L1 group exhibited a significantly greater increase in the levels of these cytokines (Figure 7H–J). The heightened cytokine levels induced by this group prove beneficial for initiating antitumor immune responses. These findings further validate that the combined treatment leveraging LP@PFH@HMME‐augmented SDT along with aPD‐L1 ICB significantly enhances the effectiveness of the antitumor immune response in the 4T1 breast cancer model.

### 2.7 In Vivo Antitumor Immune Memory Effect

**Figure 8:**
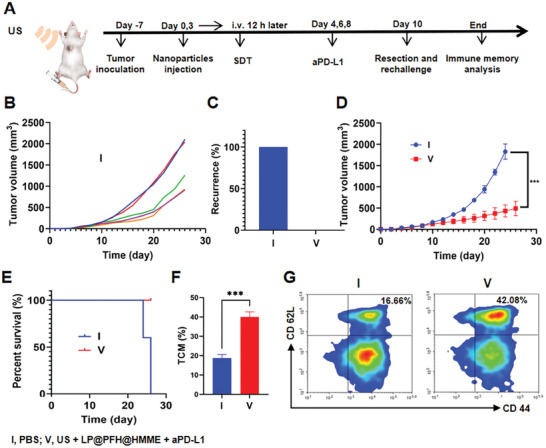
In vivo immune memory effect of LP@PFH@HMME‐augmented SDT plus aPD‐L1 therapy. A) Schematic illustration of the experimental design for recurrence and rechallenge in 4T1 tumor‐bearing mice. B) Growth curves and C) statistical data for recurrent tumors in mice receiving PBS or LP@PFH@HMME + US + aPD‐L1 treatment (*n* = 5). D) Secondary 4T1 tumor growth in mice after tumor rechallenge (*n* = 5). E) Survival time of secondary 4T1 tumor‐bearing mice after different treatments (*n* = 5, ^***^ *p* < 0.001). F) Quantitative analyses (*n* = 5, ^***^ *p* < 0.001) and G) flow cytometry analyses of central memory T cells (TCMs, CD3^+^CD8^+^CD62L^+^CD44^+^) among CD8^+^ T cells in the blood of mice that received different treatments (*n* = 3).

The immunological memory effect is a widely recognized characteristic of adaptive immunity that plays a vital role in the durable inhibition of tumors and prevention of recurrence.^[^ ^52^ ^]^ Thus, we conducted a secondary inoculation of 4T1 tumor cells into the left breast of each mouse after surgically removing the primary tumor from the right breast to further evaluate the immune memory effects of our combined tumor immunotherapy strategy (**Figure** **8**A). The recurrence of tumors after surgical resection of the primary tumor was monitored. tumor recurrence was observed in all mice receiving PBS, while no tumor recurrence was detected in mice receiving the US + LP@PFH@HMME + aPD‐L1 treatment (Figure 8B,C). Additionally, mice that underwent different treatments were rechallenged with a secondary homotypic tumor in the left breast after surgical resection of the primary tumor from the right breast. In comparison, the growth of reinoculated tumors in the combined treatment group was inhibited compared to that in the control group (Figure 8D). Compared with the control group, the survival time of mice in the combined treatment group was longer than 26 days (Figure 8E). These results demonstrated that US + LP@PFH@HMME + aPD‐L1 treatment generated long‐term immune memory effects. To assess the effect of immune memory, the proportion of central memory T cells (TCMs) was determined. TCM cells, characterized by CD3^+^, CD8^+^, CD62L^+^, and CD44^+^ expression, can survive for a long time, proliferate, and differentiate into effective memory T cells after being stimulated by tumor antigens in vivo, thus playing a crucial role in long‐term antitumor therapy.^[^ ^53^ ^]^ As shown in Figure 8F,G, the US + LP@PFH@HMME + aPD‐L1 group exhibited a significant increase in the proportion of TCMs compared to that in the control group, indicating a strong immune memory response. These results indicated that the combination of LP@PFH@HMME‐augmented SDT and immune checkpoint blockade with aPD‐L1 effectively promoted immune memory.

## 3 Conclusion

A liposome nanodrug (LP@PFH@HMME) was designed for the ^19^F MRI/CEUS bimodal imaging‐guided SDT which had the potential to enhance ICB therapy in TNBC patients. Under US irradiation, LP@PFH@HMME was triggered to produce abundant ROS and induce robust ICD in cancer cells, leading to notably promoted DCs maturation. In combination with PD‐L1 checkpoint blockade using aPD‐L1, LP@PFH@HMME under US irradiation elicited efficient antitumor immune activation and an immune memory effect to inhibit tumor growth and recurrence in TNBC. Moreover, the inclusion of the imaging agent PFH endowed the theranostic nanodrug with bimodal imaging capacity (^19^F MRI/CEUS) with a desirably long transverse relaxation time (T_2_ = 1.072 s), thus facilitating the visualization of the tumor and the accumulation of the nanodrug within the tumor site which provided a crucial guidance for SDT against the TNBC. Therefore, LP@PFH@HMME, with its multifunctionality integrating ^19^F MRI/CEUS dual imaging‐guided SDT and ICB therapy, stands out as a promising and efficient platform for the treatment of TNBC.

## 4 Experimental Section

### Materials

1,2‐Distearoyl‐sn‐glycero‐3‐phosphoethanolamine‐N‐[methoxy (poly (ethylene glycol))−2000] (DSPE‐PEG2000) and cholesterol (Chol) were purchased from AVT Pharmaceutical Tech Co., Ltd. (Shanghai, China). Phosphatidyl ethanolamine (PE) and hematoporphyrin monomethyl ether (HMME) were purchased from Macklin (Shanghai, China). 1,2‐Distearoyl‐sn‐glycero‐3‐phosphocholine (DSPC) was purchased from Aladdin (Shanghai, China). PFH was supplied by Shanghai Acmec Biochemical Co., Ltd. (Shanghai, China). Anti‐PD‐L1 was obtained from Bioxcell (aPD‐L1; clone 10 F.9G2; catalog no. BE0101). All reagents used in this work were of analytical grade and were used without further purification.

### Preparation and Characterization of the PFH‐Encapsulated/HMME‐Loaded Nanodrugs

First, DSPC:PE: Chol:DSPE‐PEG2000 at a 50:25:25:5 molar ratio (a total of 30 mg) and 100 µg of HMME were dissolved in 10 mL of trichloromethane (CHCl_3_) solution. The solution was placed on a rotary evaporator to completely remove the solvent to form a thin lipid film. The lipid film was peeled off with 5 mL of PBS (pH 7.4). Then, PFH was slowly added under sonication in an ice bath to form the PFH‐encapsulated/HMME‐loaded (LP@PFH@HMME) nanodrug. Ultimately, the LP@PFH@HMME nanodrug was harvested via an extrusion process by mini extruders using a 0.45‐µm syringe filter. The morphology and hydrodynamic particle size of the LP@PFH@HMME nanodrug were characterized by transmission electron microscopy (TEM) and dynamic light scattering (DLS), respectively. The HMME content loaded in the nanodrug was quantified using a UV–vis spectrometer.

### Relaxation Time and Quantitative NMR (qNMR)

Five hundred microliters of LP@PFH@HMME particles at a concentration of 100 mm were loaded into a nuclear magnetic tube. Relaxation time measurements were performed on a nuclear magnetic resonance (NMR) spectrometer (Bruker Avance, 600 MHz) with 10% D_2_O for shaking. Longitudinal relaxation times (T_1_) were measured using an inversion recovery (IR) sequence, and transverse relaxation times (T_2_) were measured using a CPMG sequence. The T_1_ values were obtained directly from the fitting report using the vdlist (T_1_ fitting) equation: *I*(t) = *I*(0) + *P ×* exp(− *t*/T_1_). The T_2_ values were calculated using the vclist (T_2_ fitting) equation: *I*(t) = *P ×* exp(− *t*/T_2_), where T_2_ = fitting cycle number × (2D_20_ + *P*_2_), D_20_ = 0.0005 s, and *P*_2_ = 28.7 µs.

### In Vitro ^19^F MRI and US

^1^H/^19^F MRI experiments involving nanodrugs at various concentrations were performed on a 9.4 T MRI scanner (BioSpec 94/20 USR, Bruker) equipped with commercially available ^1^H/^19^F MRI coils. The temperature was maintained at 25 °C during MRI scans. The parameters for ^1^H MRI were as follows: RARE, TR = 1000 ms; TE = 8.5 ms; slice thickness = 1 mm; field of view (FOV) = 4 × 4 cm; image matrix = 256 × 256; average = 2; and acquisition time = 1 min 36 s. The parameters for ^19^F MRI were as follows: method = RARE, TR = 800 ms; TE = 8.5 ms; slice thickness = 20 mm; FOV = 4 cm × 4 cm; image matrix = 32 × 32; average = 96; and acquisition time = 7 min 40 s 800 ms.

LP@PFH@HMME particles were exposed to US irradiation (1.0 MHz, 1.6 W cm^−2^, 50% duty cycle, 5 min) to evaluate the contrast‐enhanced ultrasound (CEUS) results of the nanodrugs with different PFH concentrations. Contrast‐enhanced ultrasound (CEUS) and power Doppler (PD) imaging of the LP@PFH@HMME particles were performed with different PFH concentrations after US irradiation and the commercial product SonoVue was used as a positive control.

### Cell Culture

The 4T1 mouse breast cancer cell line and mouse bone marrow‐derived dendritic cells (DC2.4 cells) were obtained from the American Type Culture Collection (ATCC) and cultured in RPMI 1640 medium supplemented with 1% streptomycin‐penicillin and 10% fetal bovine serum (FBS). Bone marrow‐derived cells (BMDCs) were isolated from female BALB/c mice (female, 4–6 weeks old) under sterile conditions and cultured in a 6‐well plate with RPMI 1640 medium containing 10% FBS, 10 ng mL^−1^ IL‐4, and 20 ng mL^−1^ GM‐CSF. All cells were cultured in hypoxic conditions with 5% CO_2_ at 37 °C.

### Cellular Uptake

The cellular uptake of LP@PFH@HMME particles by 4T1 cells was studied using confocal laser scanning microscopy (CLSM; Nikon C2, Japan). Briefly, 4T1 cells seeded in 12‐well plates (1 × 10^4^ cells per well) were incubated with LP@PFH@HMME particles (0.03 mm) for 1, 2, 3 and 4 h. The cells were stained with DAPI to label nuclei and observed via CLSM; subsequently, the cells were resuspended in PBS for flow cytometry.

### In Vivo ^19^F MRI and US Imaging

One hundred microliters of PFH‐loaded nano‐drug was intravenously injected into tumor‐bearing mice, and the mice were anesthetized via intraperitoneal injection of pentobarbital sodium (50 mg kg^−1^). Then, the mice were subjected to in vivo ^1^H/^19^F MRI at different time points on a 9.4T MRI scanner with the same sequence as that used for in vitro ^1^H/^19^F MRI.

One hundred microliters of PFH‐loaded nanodrug (PFH = 200 mm) was intravenously injected into the tumor‐bearing mice, and the mice were anesthetized via intraperitoneal injection of pentobarbital sodium (50 mg kg^−1^). Then, the mice were exposed to US irradiation (1.0 MHz, 1.6 W cm^−2^, 50% duty cycle, 5 min) for CEUS imaging at different time points using a Vevo 3100 high‐frequency imaging system (Fujifilm Visual Sonics) equipped with a 21‐MHz linear array transducer (MX250, Fujifilm Visual Sonics, axial resolution: 75 µm).

### Determination of ROS Generation In Vitro

In vitro, US‐triggered ROS generation by LP@PFH@HMME particles was determined with 1,3‐diphenylisobenzofuran (DPBF), which has a UV absorbance at 398 nm and is quenched by ROS. A solution containing DPBF (10 µm) and LP@PFH@HMME particles (0.03 mm) was exposed to US irradiation (1.0 MHz, 1.6 W cm^−2^, 50% duty cycle, 1, 3, 5 min), after which the UV absorbance of DPBF at 398 nm was assessed.

Next, US‐triggered ROS generation in 4T1 cells was investigated using the ROS probe 2′,7′‐dichlorofluorescein diacetate (DCFH‐DA). In brief, 4T1 cells were incubated with LP@PFH@HMME particles (0.03 mm) at 37 °C for 4 h. Afterward, the culture medium was replaced with fresh medium containing 10 µm DCFH‐DA (Beyotime) and US irradiation (1.0 MHz, 1.6 W cm^−2^, 50% duty cycle, 5 min) was applied. Next, after incubating for another 30 min, the 4T1 cells were washed with PBS three times and observed via CLSM (Nikon C2, Japan). The intracellular ROS levels were analyzed via flow cytometry (NovoCyte 3000, Agilent).

### In Vitro Cytotoxicity Assay

4T1 cells were incubated with different concentrations of the nanodrug (0, 0.01, 0.015, 0.025, 0.03, or 0.04 mm HMME and 0, 25, 50, 100, 200, or 400 mm PFH) in 96‐well plates (4 × 10^3^ cells per well) for 12 h. Then, the medium containing the nanodrug was replaced with a fresh culture medium, and the cells were exposed to US irradiation (1.0 MHz, 1.6 W cm^−2^, 50% duty cycle, 5 min). After incubation for another 12 h, the cell viability in each treatment group was measured by the CCK‐8 assay (Beyotime) to assess SDT‐induced cytotoxicity. Moreover, a live/dead cell viability assay was performed to visualize cell apoptosis after SDT. Under CLSM, living cells labeled with calcein‐AM exhibited green fluorescence, and dead cells labeled with PI exhibited red fluorescence.

### In Vitro BMDCs Maturation Assay

To explore in vitro dendritic cell (DCs) maturation induced by SDT treatment, 4T1 cells that received different treatments were cultured in the upper compartment of a transwell system, and bone marrow‐derived cells (BMDCs) isolated from female BALB/c mice (4–6 weeks) were seeded in the lower compartment. After 24 h of incubation, the BMDCs were collected and stained with FITC‐conjugated anti‐CD11c (Tonbo Bioscience, Catalog: 35–0114), Brilliant Violet 421‐conjugated anti‐CD80 (Biolegend, Catalog: 104 726) and PE‐conjugated anti‐CD86 (Biolegend, Catalog: 105 008) for flow cytometry analysis (NovoCyte 3000, Agilent).

Furthermore, the morphology of the mature DCs was observed via CLSM. In brief, DC2.4 cells were seeded in confocal dishes (1 × 10^4^ cells per well) for 12 h and then subjected to different treatments. After 24 h, the cells were fixed with 4% paraformaldehyde and then incubated with phalloidin (red) for 1 h. Then, the cells were stained with DAPI and observed via CLSM (Nikon C2, Japan).

### Tumor Growth Inhibition

First, the accumulation of the nano‐drug in tumors was explored in a breast tumor model. In brief, DiR‐loaded nano drugs were intravenously injected into 4T1 tumor‐bearing mice, and in vivo and *ex vivo* fluorescence imaging was subsequently performed on a Carestream Multispectral FX Pro in vivo Imager at different time points.

Next, the antitumor effect of the nanodrug was assessed. When the tumor volume reached ≈100 mm^3^ at 7 days postinoculation, the tumor‐bearing mice were randomly divided into 5 groups (PBS, LP@PFH@HMME, aPD‐L1, LP@PFH@HMME + US, and LP@PFH@HMME + US + aPD‐L1). The nanodrug (HMME dose: 2 mg kg^−1^ body weight) was intravenously injected into 4T1 tumor‐bearing mice; the nanodrug was administered twice (day 0 and day 3). Then, the tumor sites within the mice were exposed to US irradiation (1.0 MHz, 1.6 W cm^−2^, 50% duty cycle, 5 min) 12 h post‐injection. Next, aPD‐L1 (2.5 mg kg^−1^ body weight) was administered every two days 3 times (Day 4, Day 6, and Day 8). The tumor size and mouse body weight were recorded every two days. The tumor volume was calculated as follows: 0.5 × length × width.^2^

### In Vivo Immune Memory Effects

Subcutaneous 4T1 tumors were established on the right breast of each mouse. The tumor‐bearing mice were randomly divided into 5 groups (PBS, LP@PFH@HMME, aPD‐L1, LP@PFH@HMME + US, and LP@PFH@HMME + US + aPD‐L1). Then, the primary tumors were removed via surgical resection. Three days later, a secondary homotypic tumor was established on the left breast of each mouse. The recurrence and growth of the secondary homotypic tumor were monitored.

### In Vivo Immunological Effects of SDT

On day 10 after treatment, the tumor tissues and lymph nodes from each group of mice were harvested to prepare single‐cell suspensions for flow cytometry (NovoCyte 3000, Agilent). The tumor cell suspensions and the lymph node cells were stained with an antibody cocktail for T‐cell detection (APC‐conjugated anti‐CD3 (BioLegend, Catalog: 100 236), FITC‐conjugated anti‐CD4 (BioLegend, Catalog: 100 406) and PE‐conjugated anti‐CD8a (BioLegend, Catalog: 100 708)) and DCs detection (FITC‐conjugated anti‐CD11c (Tonbo Bioscience, Catalog: 35–0114), PE‐conjugated anti‐CD80 (BioLegend, Catalog: 104 707) and APC‐conjugated anti‐CD86 (BioLegend, Catalog: 105 011)).

### Histological Staining

The tumors, as well as the main organs (heart, liver, spleen, lungs, and kidneys), were harvested to prepare paraffin and frozen sections. Then, the prepared paraffin and frozen sections were subjected to hematoxylin‐eosin (H&E) staining, TdT‐mediated dUTP nick end labeling (TUNEL) staining, and immunohistochemical staining.

### Statistical Analysis

Statistical analysis was performed using an unpaired Student's *t*‐test or one‐way ANOVA with Tukey's test (GraphPad Prism, La Jolla, CA). The data are expressed as the mean ± standard deviation (SD), and *p* < 0.05 was considered to indicate statistical significance.

## Conflict of Interest

The authors declare no conflict of interest.

## Supporting information

Supporting Information

## Data Availability

The data that support the findings of this study are available from the corresponding author upon reasonable request.
