# Correction to “^19^F MRI/CEUS Dual Imaging‐Guided Sonodynamic Therapy Enhances Immune Checkpoint Blockade in Triple‐Negative Breast Cancer”

**DOI:** 10.1002/advs.77251

**Published:** 2026-08-19

Q. Chen, H. Xiao, L. Hu, Y. Huang, Z. Cao, X. Shuai, Z. Su, “^19^F MRI/CEUS Dual Imaging‐Guided Sonodynamic Therapy Enhances Immune Checkpoint Blockade in Triple‐Negative Breast Cancer,” *Advanced Science* 11, no. 36 (2024): 2401182, https://doi.org/10.1002/advs.202401182.

In Figure 2H, the images of the LP@HMME group at 0 and 24 h were inadvertently duplicated from those of the LP@PFH@HMME group. In Figure 4A, the images for the LP@PFH@HMME group were mistakenly duplicated from the PBS group. The corrected Figures 2H and 4A and corresponding figure legend are provided.

**Corrected Figure 2H**:


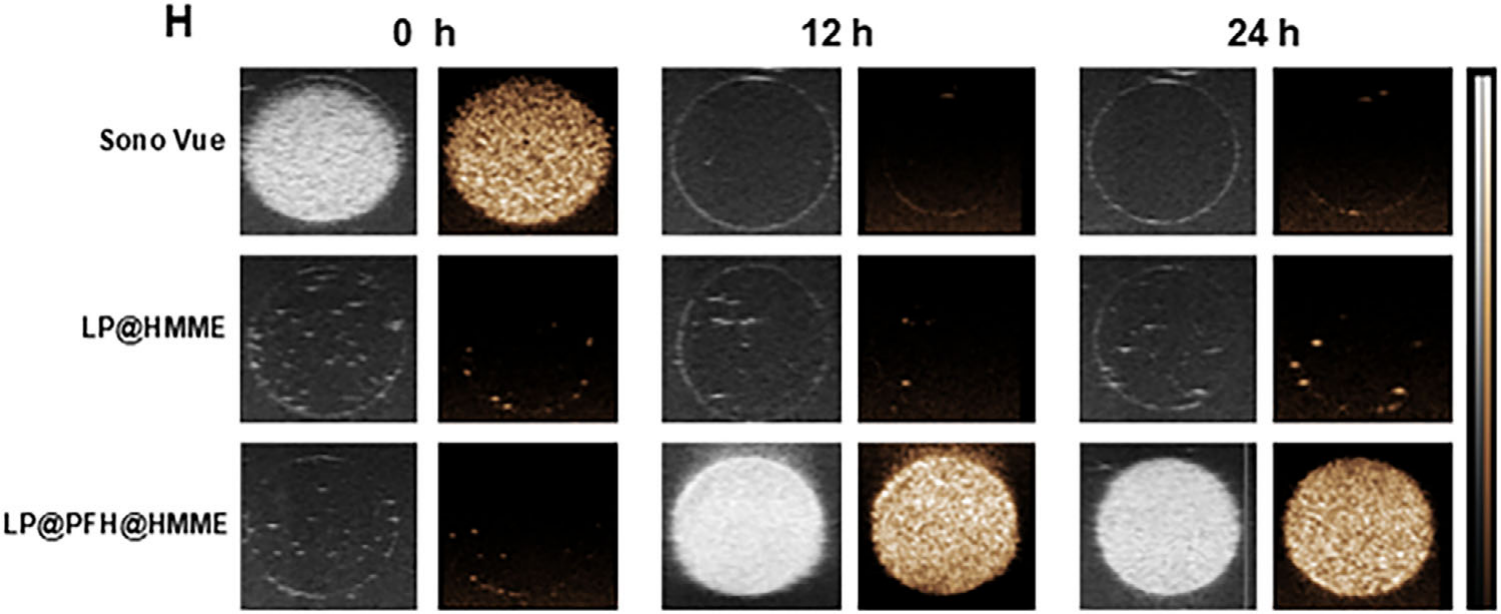


**FIGURE 2** | Characterization of the nanodrug. (H) US imaging capacity of LP@PFH@HMME after growing to microbubbles under US irradiation (1.0 MHz, 1.6 W/cm^2^, 50% duty cycle, 5 min). SonoVue and LP@HMME were used as controls. (Left, grayscale; right, harmonic in each pair of images).

**Corrected Figure 4A**:


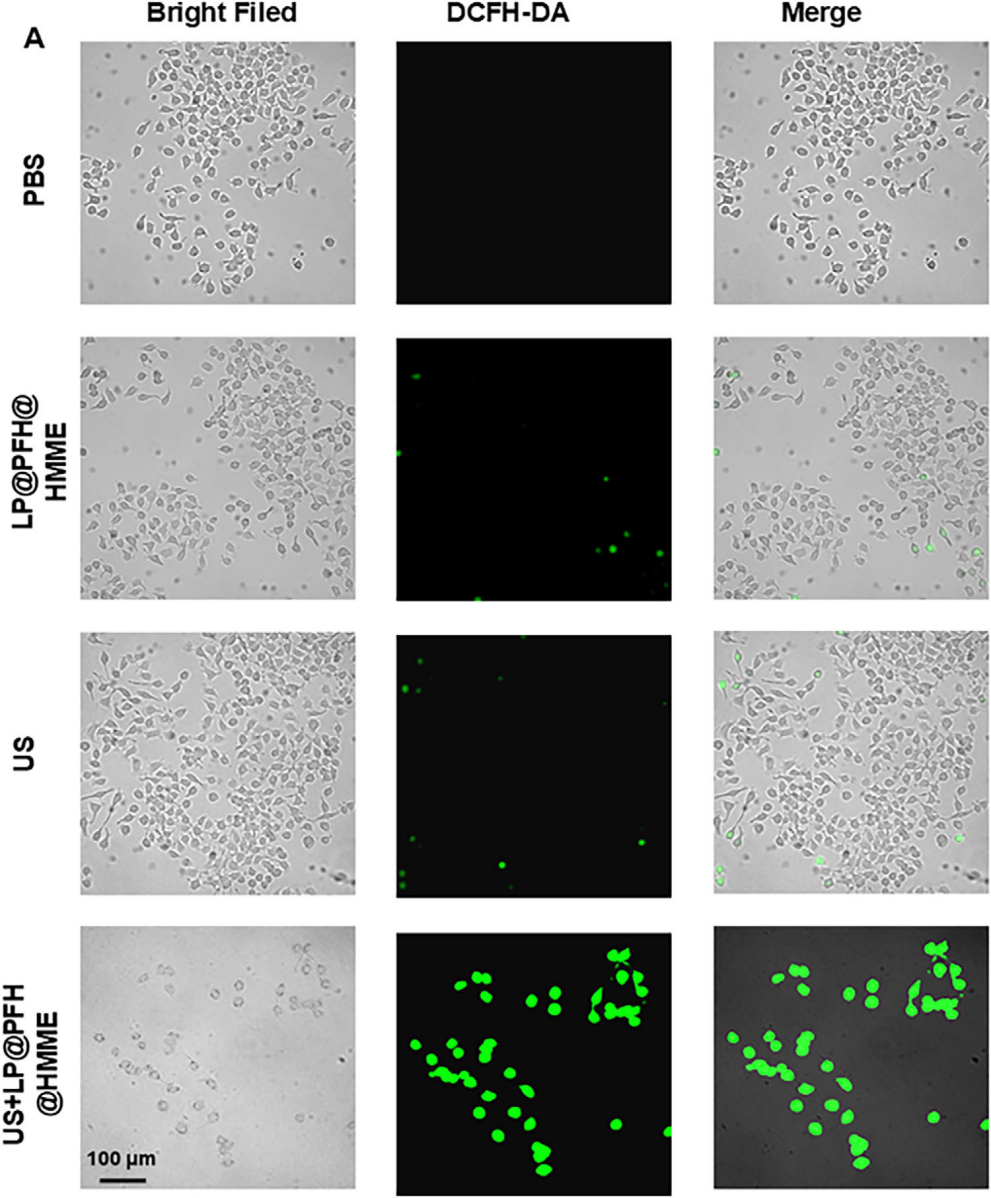


**FIGURE 4** | In vitro ROS generation by LP@PFH@HMME in 4T1 cells upon US irradiation and DC maturation. (A) CLSM images of 4T1 cells showing ROS generation detected by DCFH‐DA.

**A note added to the figure caption of 6B**:

**FIGURE 6B**. In vivo tumor growth curves against time for 4T1 tumor‐bearing mice receiving different treatments (n = 5, ^***^ *p* < 0.001). Tumor length and width were measured by a caliper and the volume was calculated using the formula *V = 0.5 × length × width^2^*. As a measurement directly taken on live mice with the tumors still beneath the skin, the results are affected by multiple factors such as fur, skin, and adjacent tissue.

These corrections and added note do not affect the findings and conclusions of the paper.

We sincerely apologize for the errors and unclear caption.

