# Supplementary material for: 19F MRI/CEUS Dual Imaging‐Guided Sonodynamic Therapy Enhances Immune Checkpoint Blockade in Triple‐Negative Breast Cancer
Source: Adv Sci (Weinh). 2024 Jul 25;11(36):2401182. doi: 10.1002/advs.202401182 (PMC11423248; doi:10.1002/advs.202401182)
Supplement: Supplementary file 1 — Supporting Information [file ADVS-11-2401182-s001.docx]

# ^19^F MRI/CEUS Dual Imaging-Guided Sonodynamic Therapy Enhances Immune Checkpoint Blockade in Triple-Negative Breast Cancer

*Qiu Chen,^1,#^* *Hong Xiao,*^3,#^ Lijun Hu**,^1^ Yongquan Huang,^1^ Zhong Cao,*^4,5^ Xintao Shuai,*^2^* *Zhongzhen Su*^1^*

^1^ Department of Ultrasound, The Fifth Affiliated Hospital, Sun Yat-Sen University, Zhuhai 519000, P. R. China.

^2^ Nanomedicine Research Center, The Third Affiliated Hospital of Sun Yat-sen University, Guangzhou 510630, China

^3^ Department of Medical Ultrasonic, The Third Affiliated Hospital of Sun Yat-sen University, Guangzhou 510630, China

^4^ School of Biomedical Engineering, Shenzhen Campus of Sun Yat-sen University, Shenzhen, Guangdong, 518107, China

^5^ Shenzhen International Institute for Biomedical Research, Longhua District, Shenzhen 518116, Guangdong, China

^#^Q.C. and H.X. contributed equally to this work.

* Correspondence should be addressed to:

Zhongzhen Su

E-mail: suzhzh3@mail.sysu.edu.cn

Xintao Shuai

E-mail: shuaixt@mail.sysu.edu.cn

Zhong Cao

E-mail: caozhong@mail.sysu.edu.cn

**Supplementary Figures**


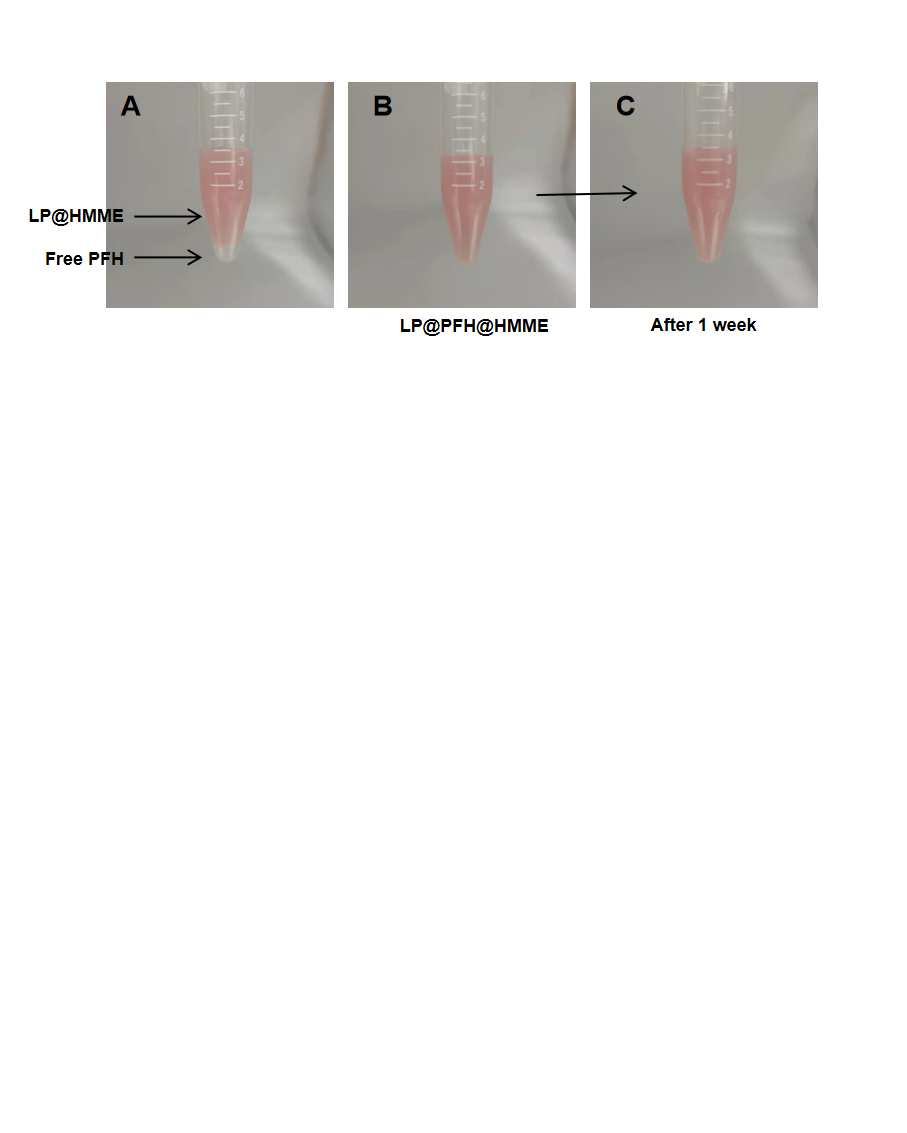


**Figure S1.** (A) Photos of mixed solutions of LP@HMME and free PFH. (B) Photos of LP@PFH@HMME in PBS. (C) Photos of LP@PFH@HMME after 1 week at 4℃.


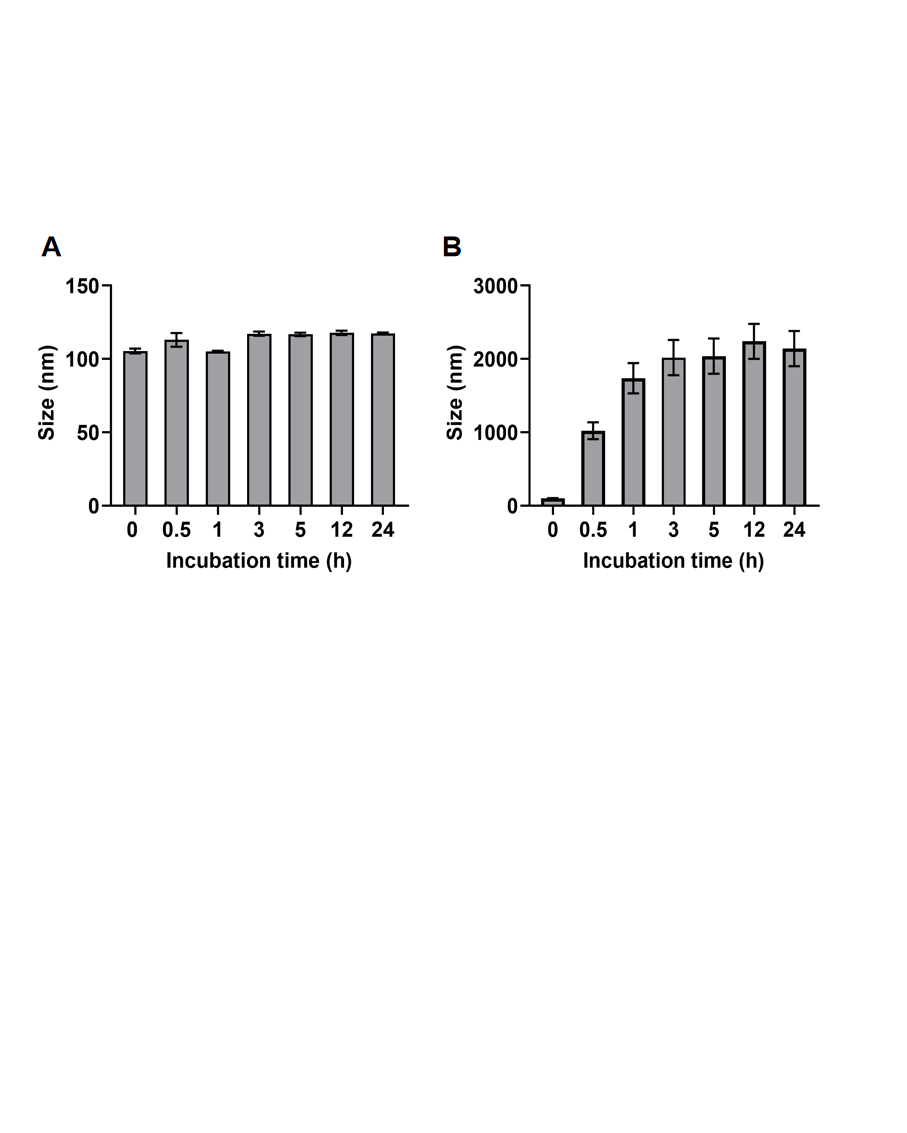


**Figure S2.** (A) LP@PFH@HMME size changes with time at 37 ℃ and pH 7.4 solutions. (B) Particle underwent a “nano-to-micro” transformation of LP@PFH@HMME dispersed at 37 ℃ and pH 6.5 solutions.


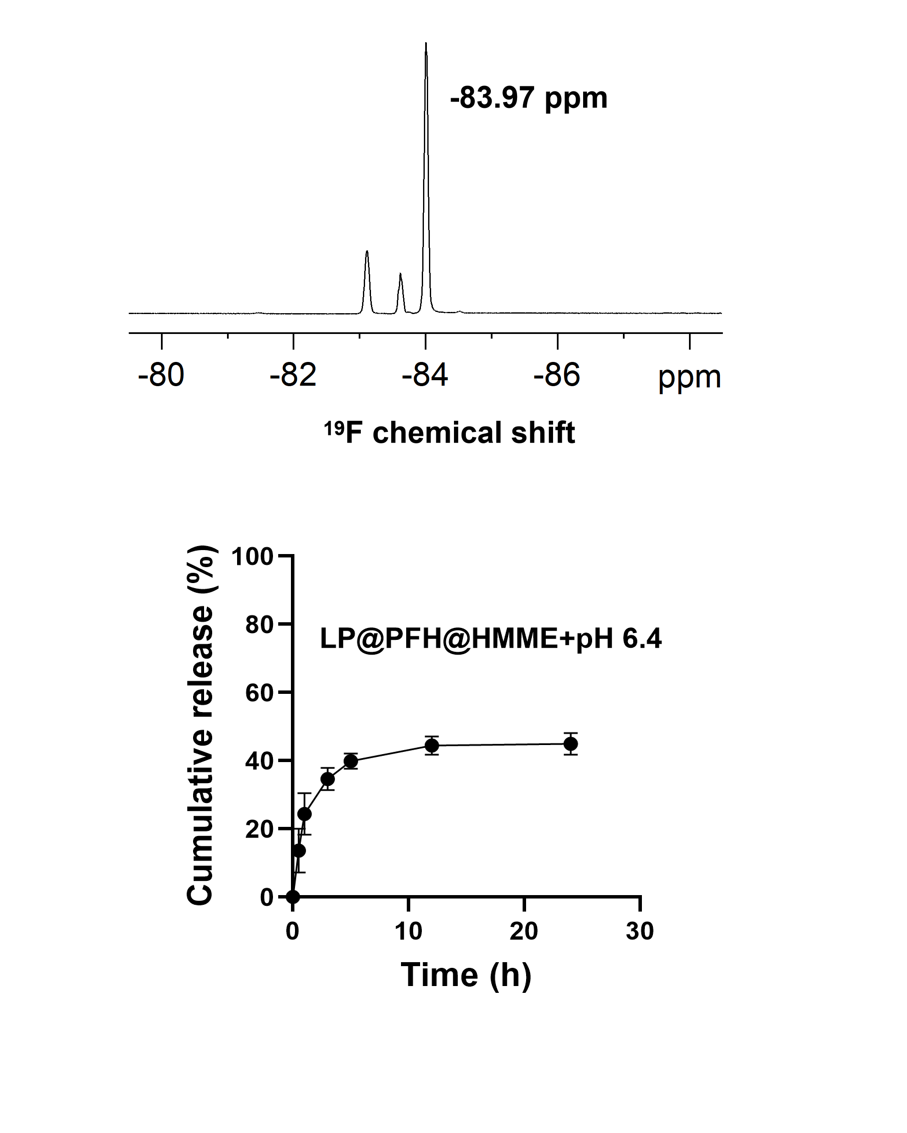


**Figure S3.** The quantitative cumulative release rate of HMME from LP@PFH@HMME in pH 6.4 during 24 hours. Data are showed as mean ± SD (n = 3)


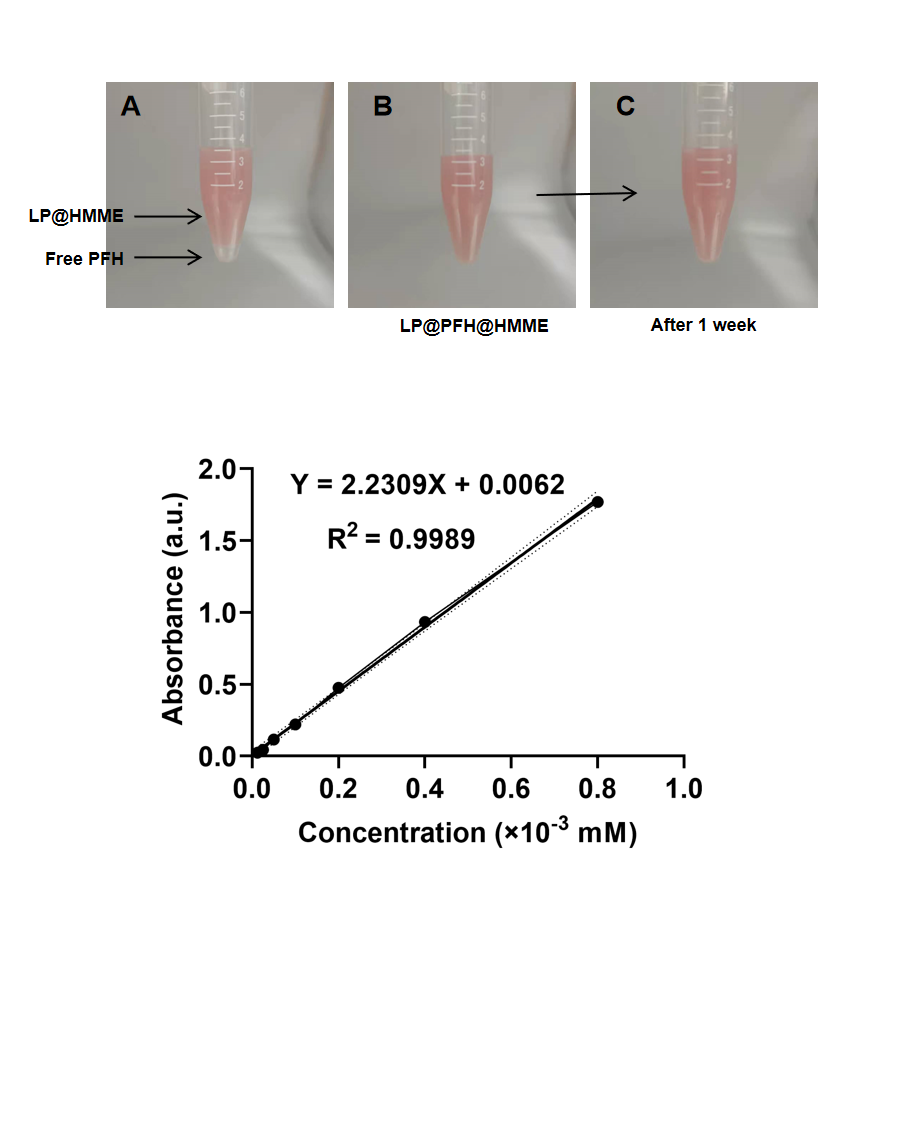


**Figure S4.** The standard curve of HMME in PFH@PFH@HMME at the wavelength of 398 nm.


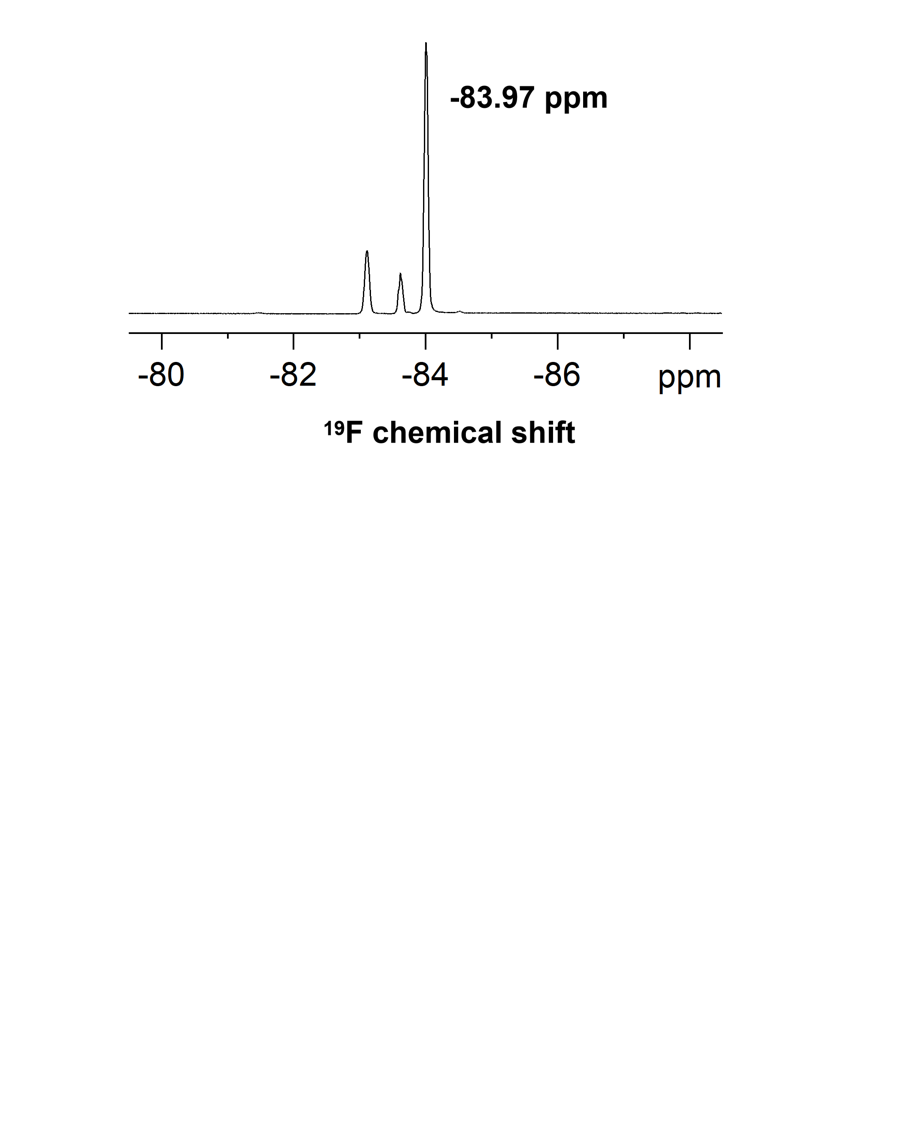


**Figure S5.** ^19^F NMR spectrum of LP@PFH@HMME.


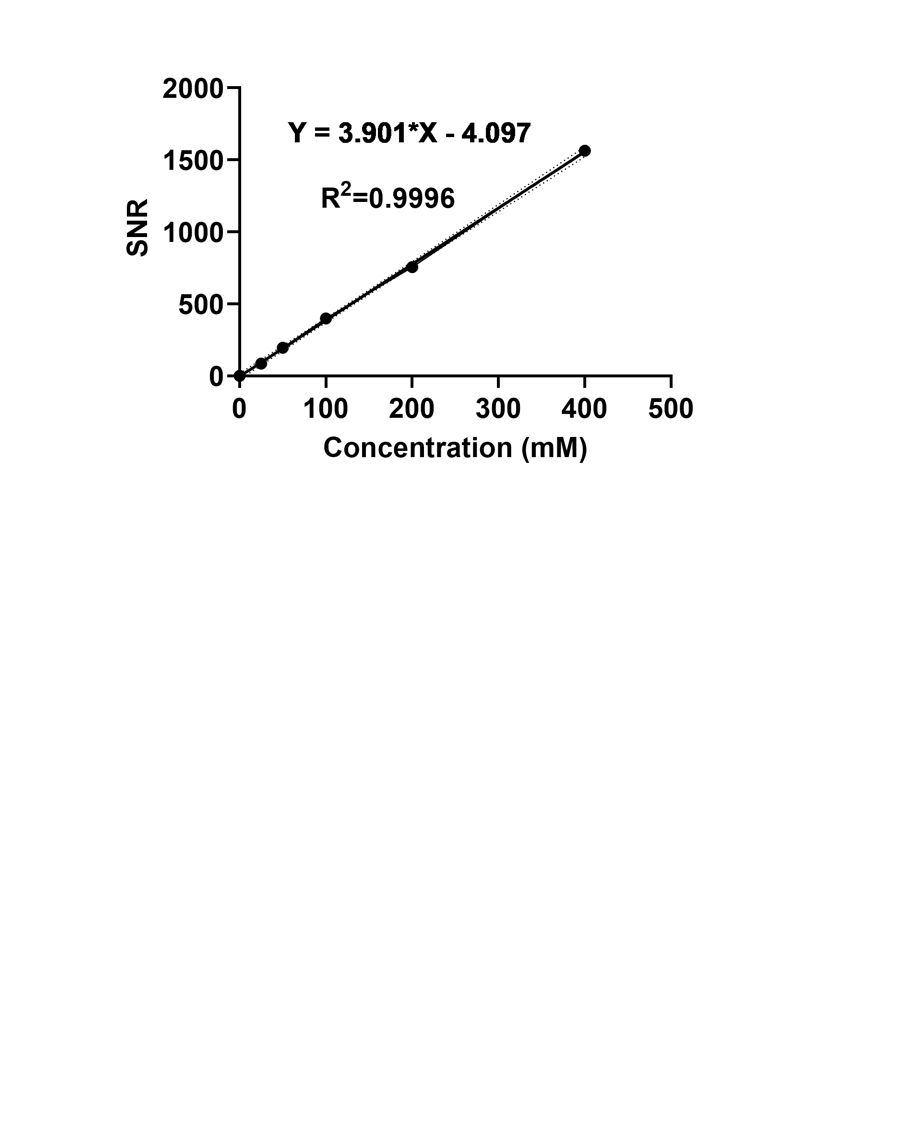


**Figure S6.** Plot of the ^19^F MRI signal-to-noise ratio (SNR) *vs.* LP@PFH@HMME concentration.


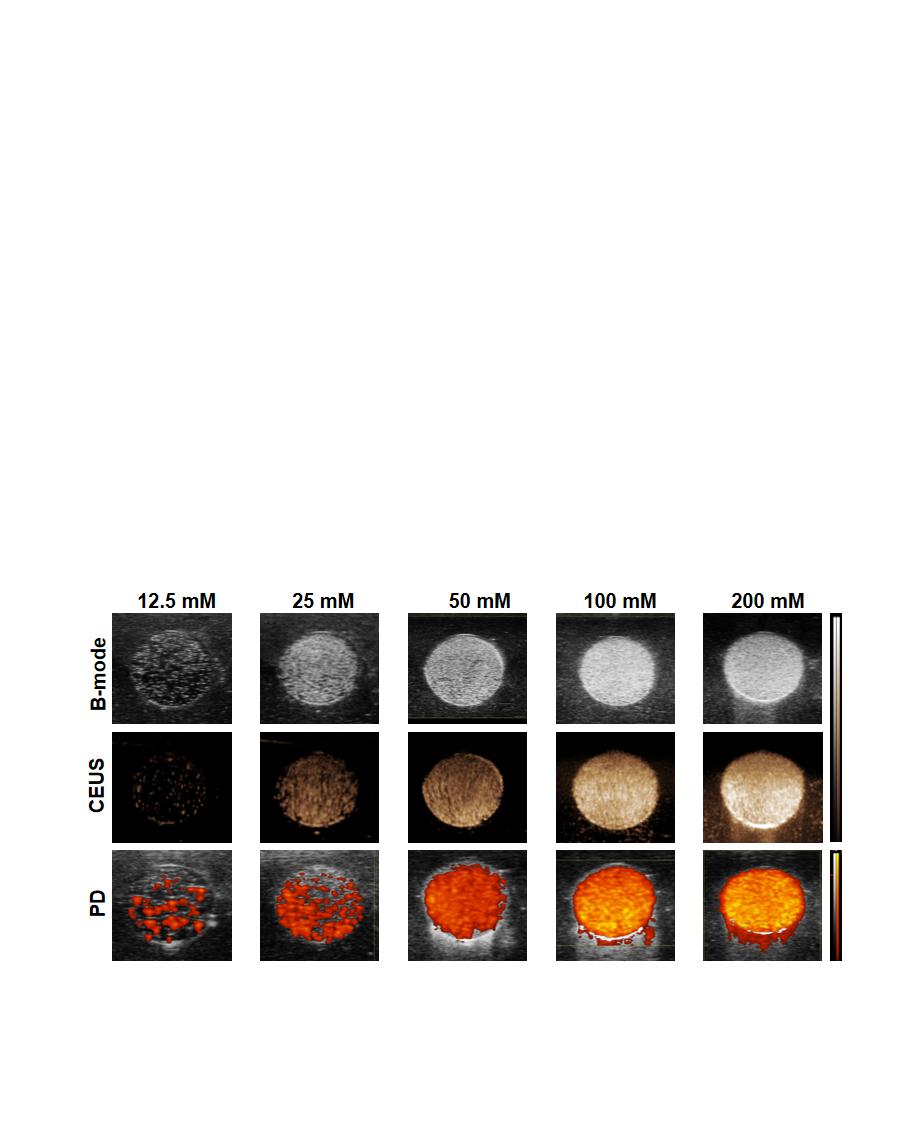


**Figure S7.** US images (up, grayscale; middle, CEUS; down, PD) of LP@PFH@HMME at various concentrations.


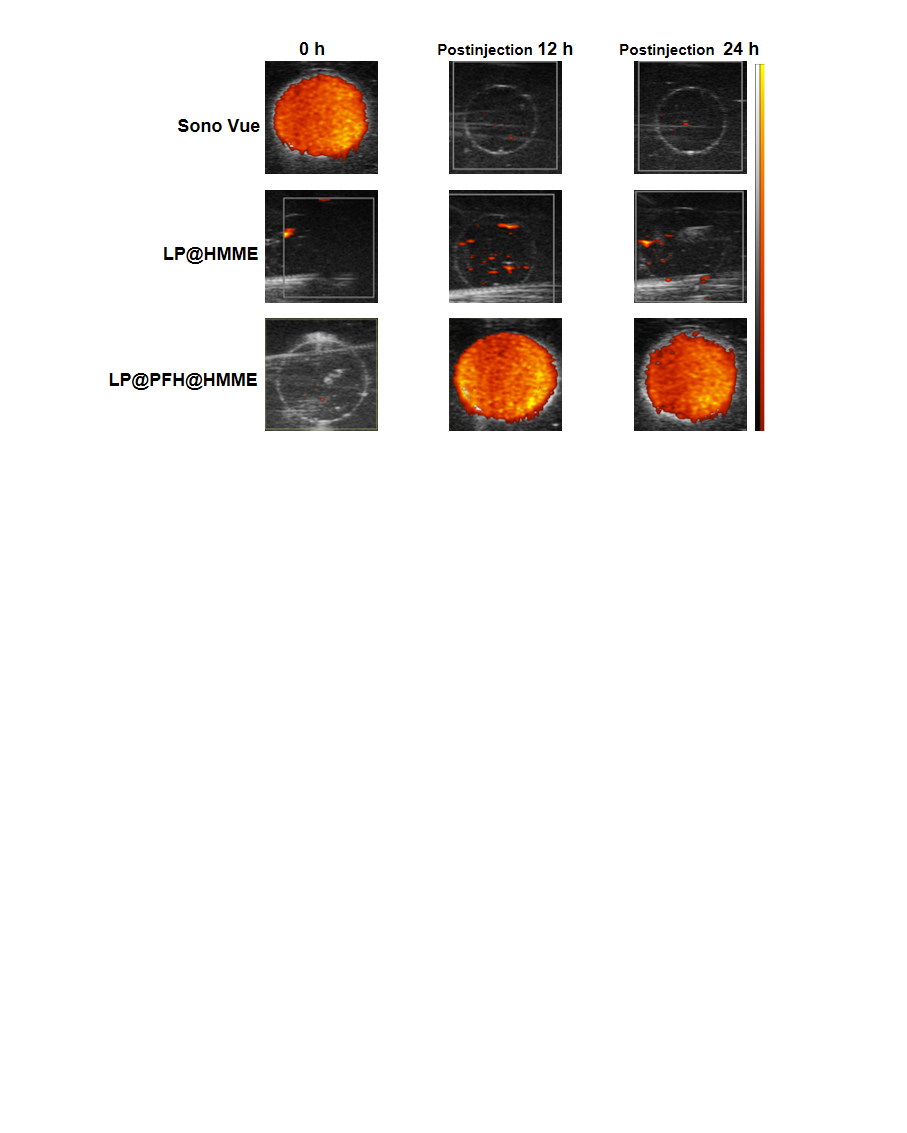


**Figure S8.** The power Doppler (PD) of LP@PFH@HMME after growing to microbubbles under US exposure irradiation (1.0 MHz, 1.6 W/cm^2^, 50% duty cycle, 5 min). SonoVue and LP@HMME were used as controls.


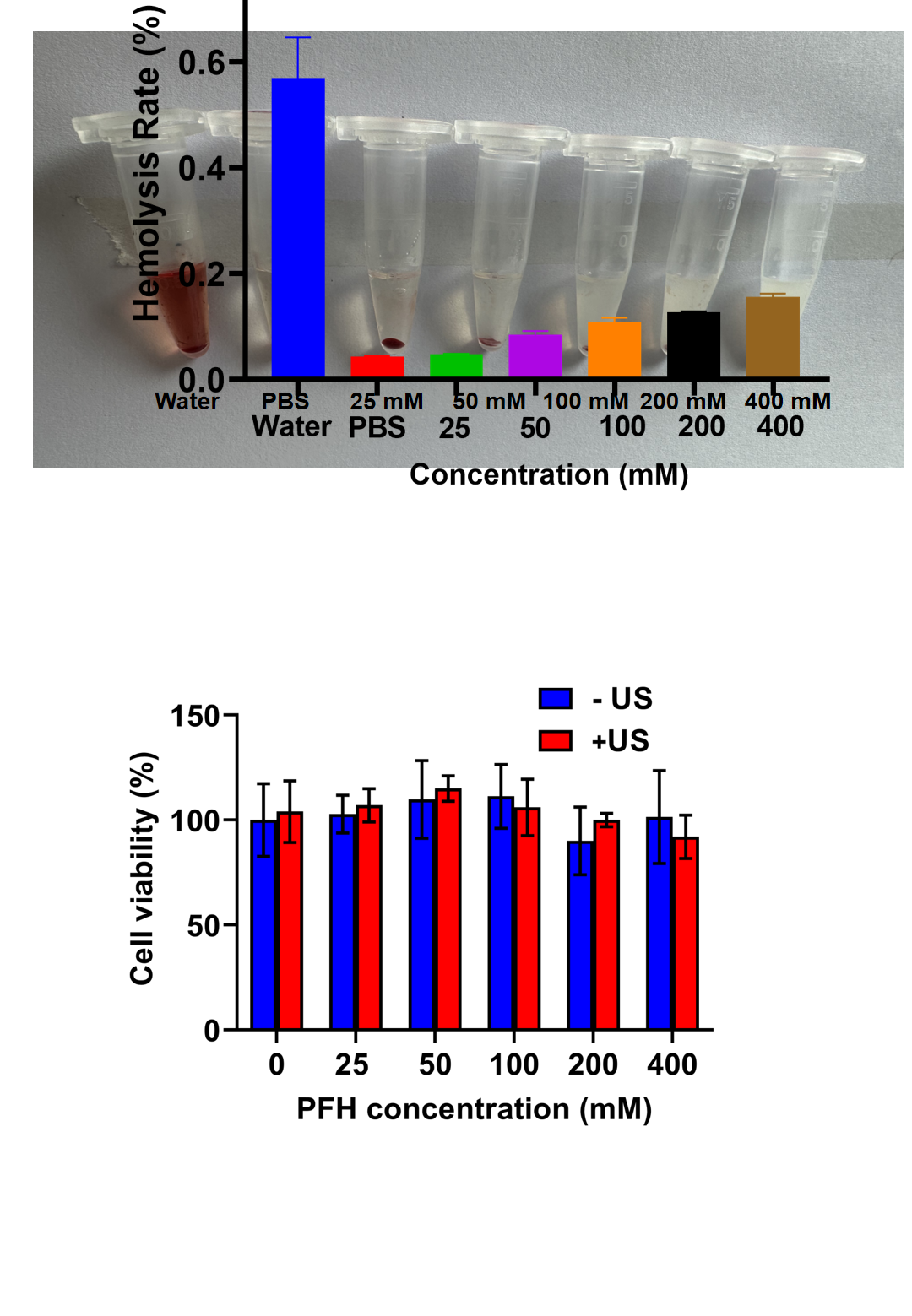


**Figure S9.** Cell viability of 4T1 cells incubated with LP@PFH for 12 h at various concentrations with/without US irradiation (US, 1.0 MHz, 1.6 W/cm^2^, 50% duty cycle, 5 min) (n = 3).


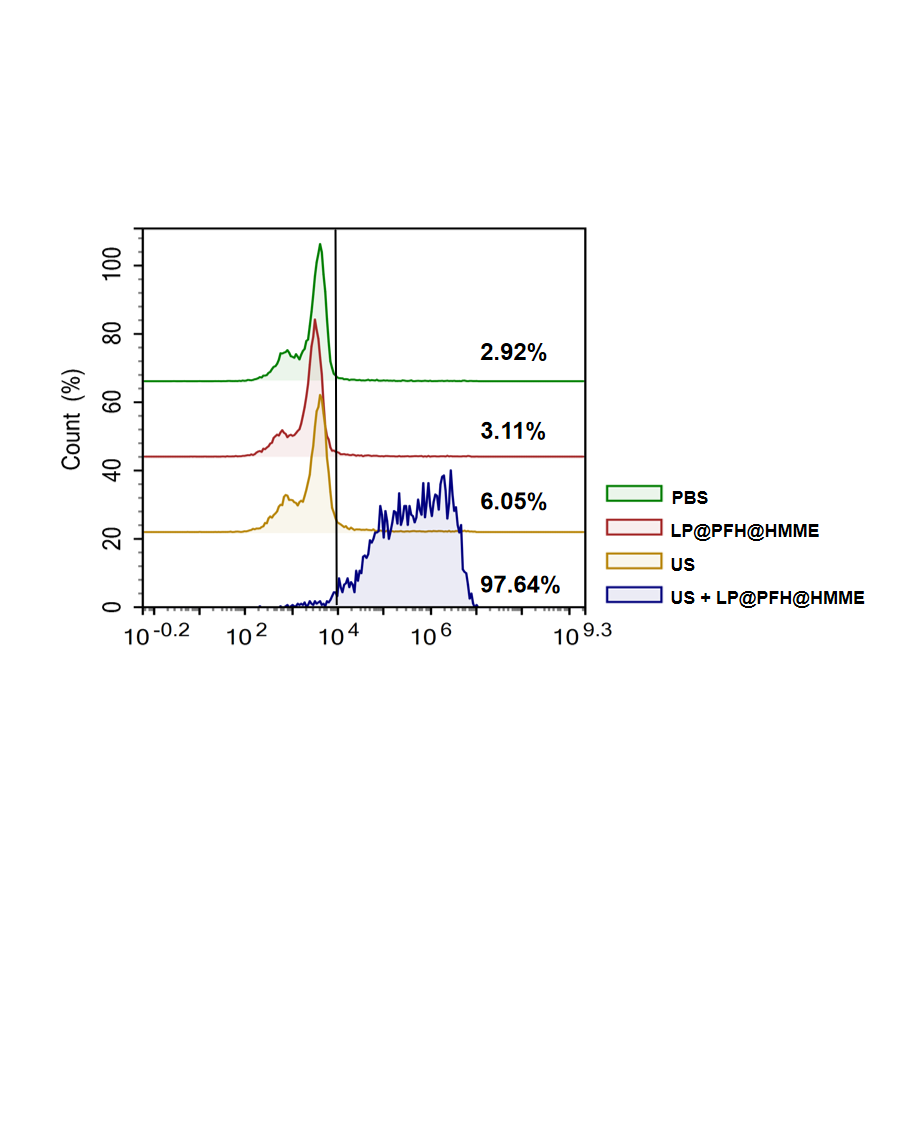


**Figure S10.** ROS generation in 4T1 cells receiving different treatments determined by flow cytometry.


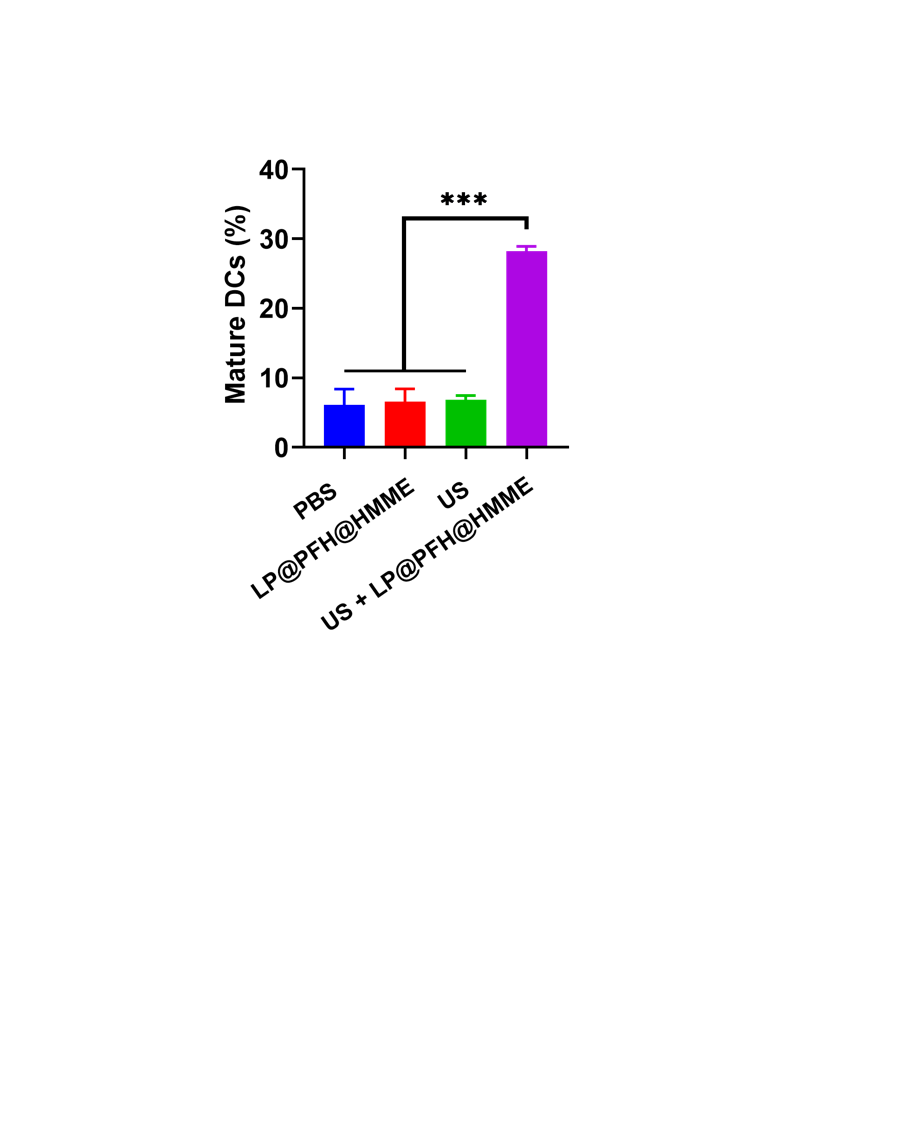


**Figure S11.** The proportions of CD80^+^CD86^+^ cells among CD11c^+^ BMDCs were analysed via flow cytometry (n = 3).


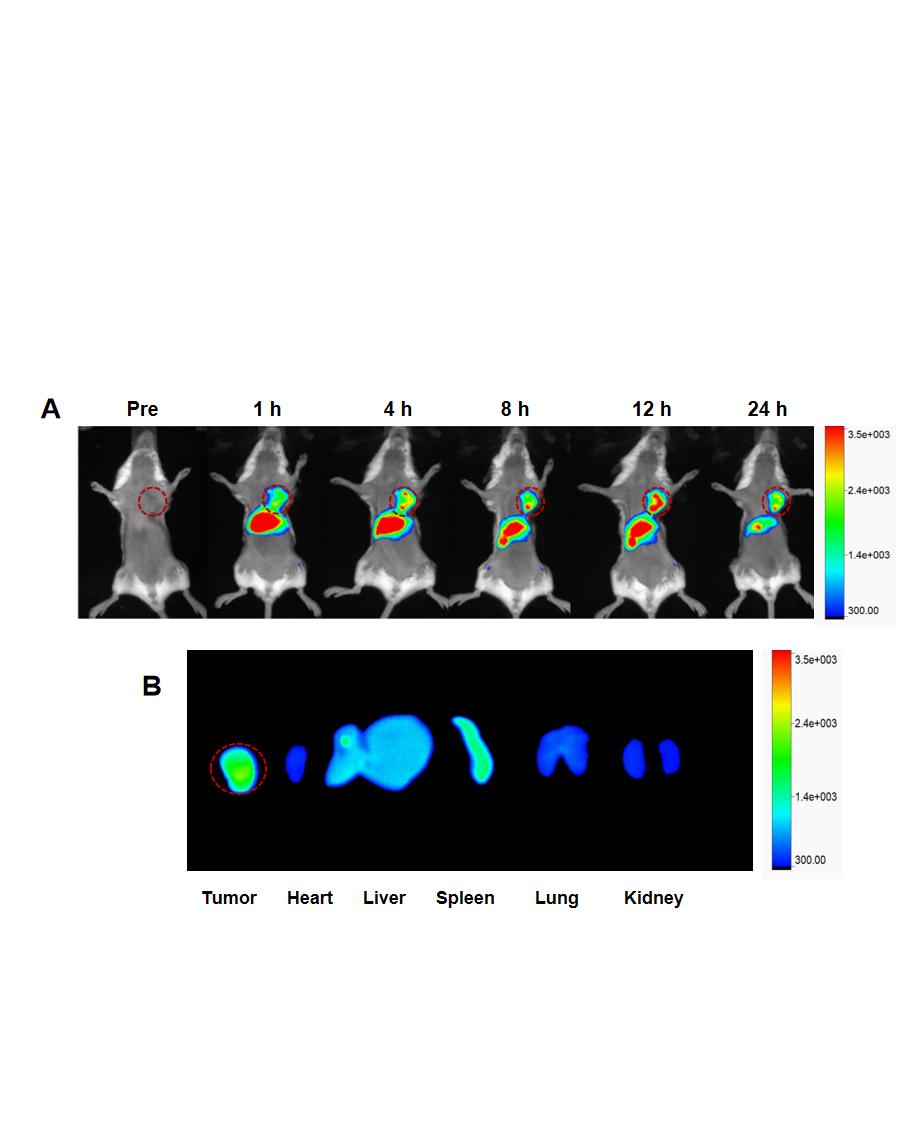


**Figure S****12.** (A) *In vivo* fluorescence imaging revealing the biodistribution of LP@PFH@HMME in 4T1 tumor-bearing mice at the indicated time points. (B) *Ex vivo* fluorescence image of the major organs and tumor tissues at 24 h post-injection.


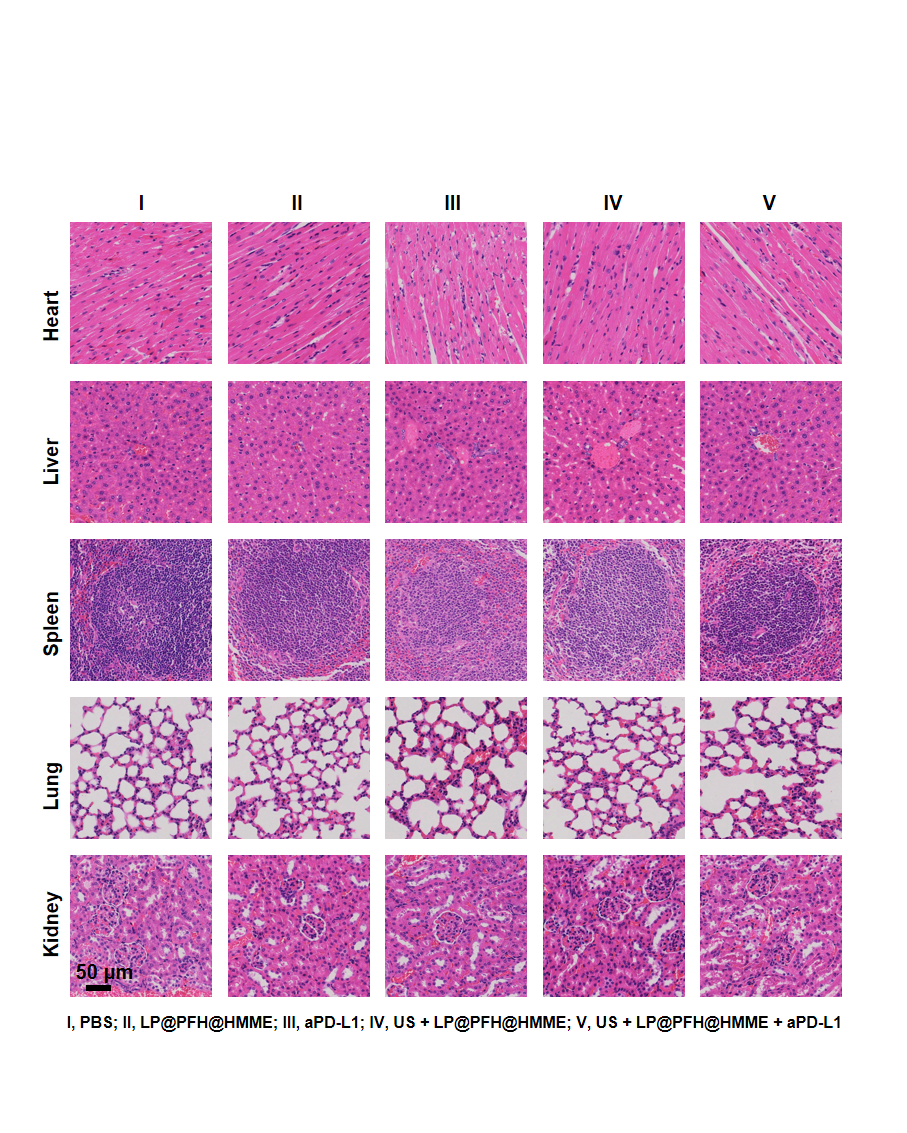


**Figure S13.** H&E staining of the major organ tissues from the mice receiving different treatments (scale bar = 50 μm).


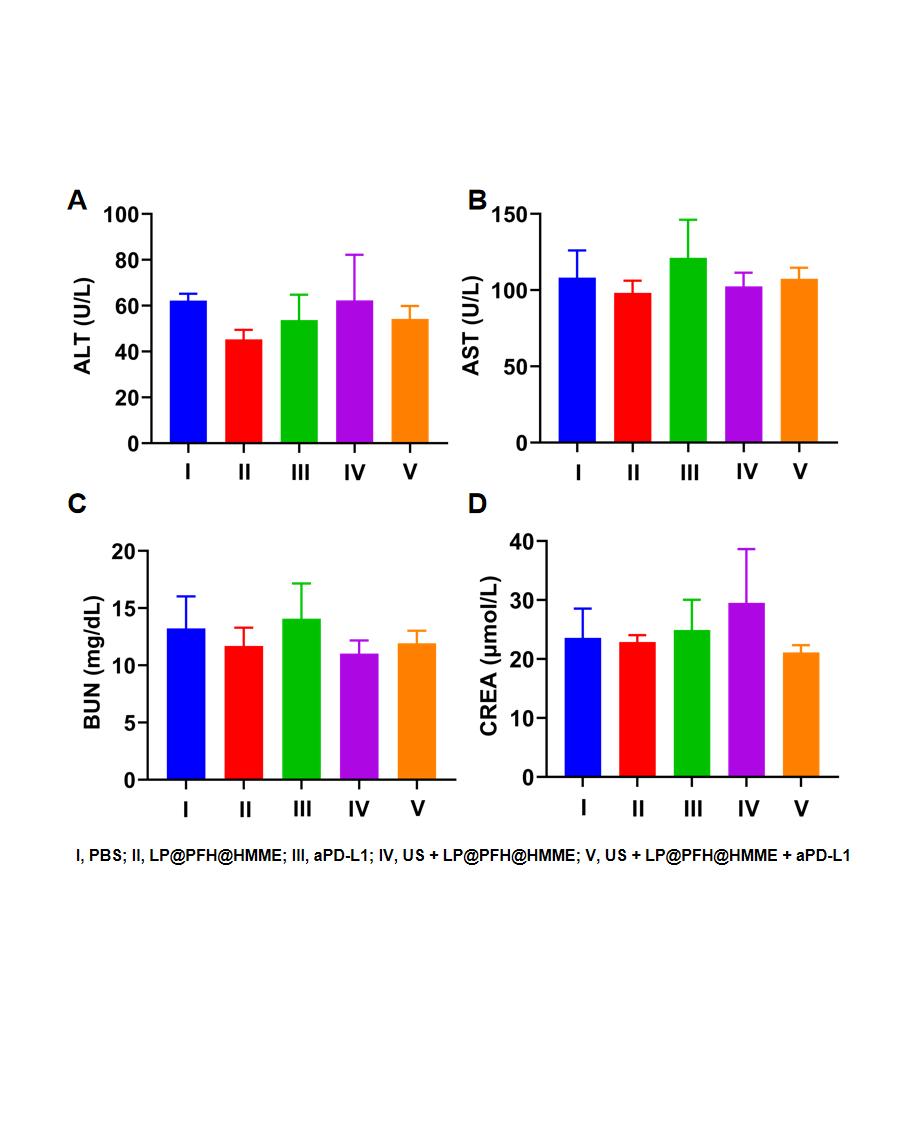


**Figure S14.** The serum levels of ALT (A), AST (B), BUN (C) and CREA (D) in 4T1 tumor-bearing mice receiving different treatments (n = 4). Healthy mice without any treatment were used as the control.


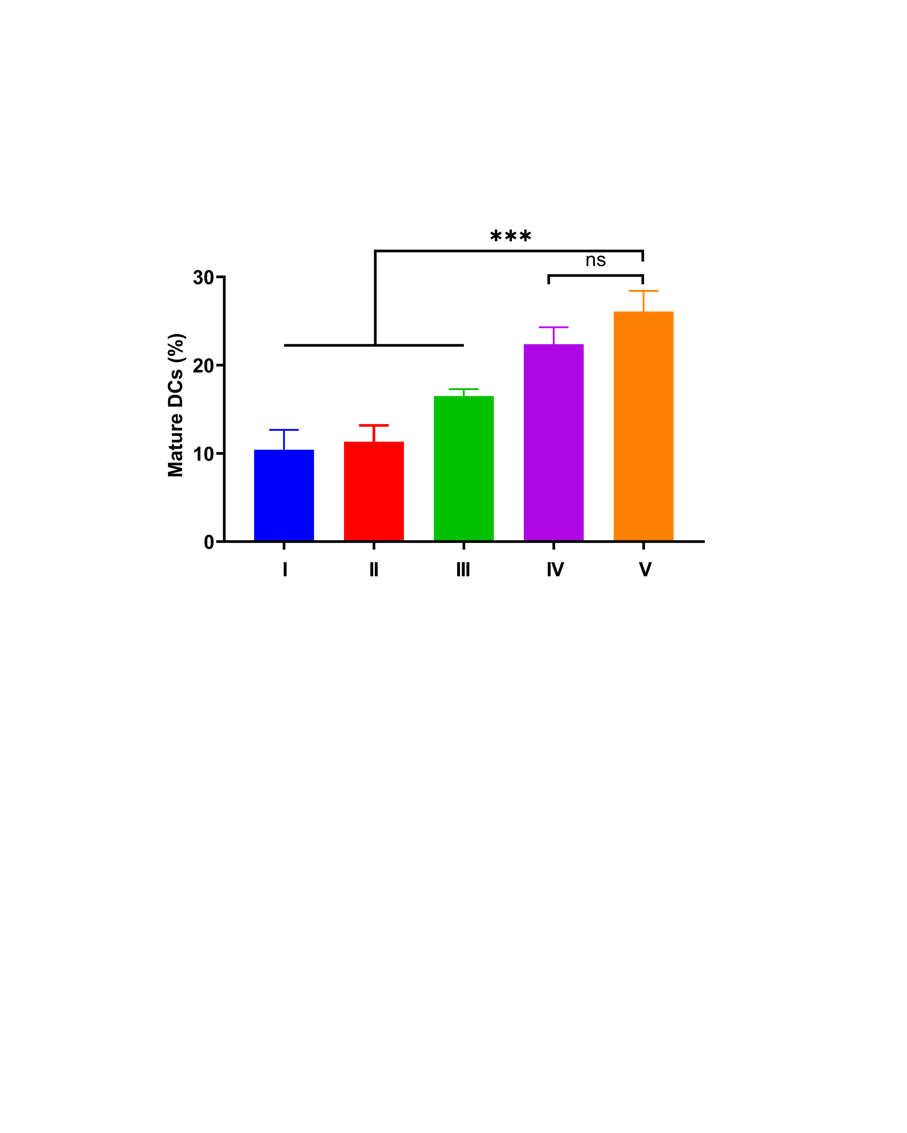


**Figure S15.** Quantitative statistics of mature BMDCs (CD80^+^CD86^+^) in different treatment groups.
